# Mechanistic insights into plasmonic photocatalysts in utilizing visible light

**DOI:** 10.3762/bjnano.9.59

**Published:** 2018-02-19

**Authors:** Kah Hon Leong, Azrina Abd Aziz, Lan Ching Sim, Pichiah Saravanan, Min Jang, Detlef Bahnemann

**Affiliations:** 1 Department of Environmental Engineering, Faculty of Engineering and Green Technology, Universiti Tunku Abdul Rahman, Jalan Universiti, Bandar Barat, 31900, Kampar, Perak, Malaysia; 2 Faculty of Engineering Technology, Universiti Malaysia Pahang, Lebuhraya Tun Razak, Gambang, 26300 Kuantan, Pahang, Malaysia; 3 Environmental Nanotechnology Laboratory, Department of Environmental Science and Engineering, Indian Institute of Technology (ISM) Dhanbad 826004, Jharkhand, India; 4 Department of Environmental Engineering, Kwangwoon University, 447-1 Wolgye-Dong, Nowon-Gu, Seoul, South Korea; 5 Institut für Technische Chemie, Leibniz Universität Hannover, Callinstrasse 3, 30167 Hannover, Germany

**Keywords:** localized surface plasmon resonance (LSPR), noble metal, plasmonic photocatalyst, reactive radicals, Schottky junctions, visible light

## Abstract

The utilisation of sunlight as an abundant and renewable resource has motivated the development of sustainable photocatalysts that can collectively harvest visible light. However, the bottleneck in utilising the low energy photons has led to the discovery of plasmonic photocatalysts. The presence of noble metal on the plasmonic photocatalyst enables the harvesting of visible light through the unique characteristic features of the noble metal nanomaterials. Moreover, the formation of interfaces between noble metal particles and semiconductor materials further results in the formation of a Schottky junction. Thereby, the plasmonic characteristics have opened up a new direction in promoting an alternative path that can be of value to the society through sustainable development derived through energy available for all for diverse applications. We have comprehensively prepared this review to specifically focus on fundamental insights into plasmonic photocatalysts, various synthesis routes, together with their strengths and weaknesses, and the interaction of the plasmonic photocatalyst with pollutants as well as the role of active radical generation and identification. The review ends with a pinnacle insight into future perspectives regarding realistic applications of plasmonic photocatalysts.

## Review

### Introduction

Photocatalysts have played and will continue to play a pivotal role in environmental and energy applications in order to fulfil the needs of the current and future generation. They cleverly tackle the various limitations in the aforementioned field to satisfy the requirements of clean and green energy, and sustainable treatment of water and air. Indeed, years of research have been directed towards developing this sustainable process for a profound, promising and reliable approach towards energy generation and environmental remediation [1–2]. In line with this, the identification of photocatalysts capable of harvesting energy from the wider electromagnetic spectrum has become the focus of most researchers. The ability to harvest such a wide spectrum will lead to a pathway for better utilization of the solar spectrum. The invention and progression of plasmonic photocatalysts laid a foundation for the successful utilisation of longer wavelengths, known as “visible light photocatalysis”.

The localised surface plasmon resonance (LSPR) is a unique characteristic of a plasmonic material, which can extend the absorption of light towards the visible light spectrum. Thus, LSPR greatly supports the utilisation of the solar spectrum, which comprises a considerable portion (≈43%) of these wavelengths. The resonance appears when the photons interact with the metal nanoparticle surface conduction electrons [3]. This phenomenon enables these photocatalysts to concentrate the light energy surrounding it and leads to a strong improvement and activation of electron movement within the metal (i.e, noble metals) and semiconductor material [4].

Besides this, another distinguishing characteristic of plasmonic photocatalysts is that they also behave as an electron trap. The incorporation of a noble metal with semiconductors in the formation of Schottky junctions contributes to this behaviour [5]. This barrier formation prevents the recombination of electrons with the holes at the valance bands by trapping the electrons excited to the conduction bands. The LPSR, along with this distinctive characteristic feature, strengthens and contributes to the improvement of the plasmonic photocatalyst.

All these distinctive characteristics of plasmonic photocatalysts have motivated researchers in the field of light-driven nanomaterials, resulting in abundant findings on the successful application of this phenomenon through the support of noble metal. Hence, we deemed a comprehensive and timely review on this topic beneficial to its development, especially in the context of promoting sustainable photocatalysts. This review exclusively discusses the recent advances with regards to synthesis, the mechanism behind the plasmonic phenomenon, quantum efficiency, identification of active radicals and future perspectives.

### Fundamentals of plasmonic photocatalysts

**Figure 1:**
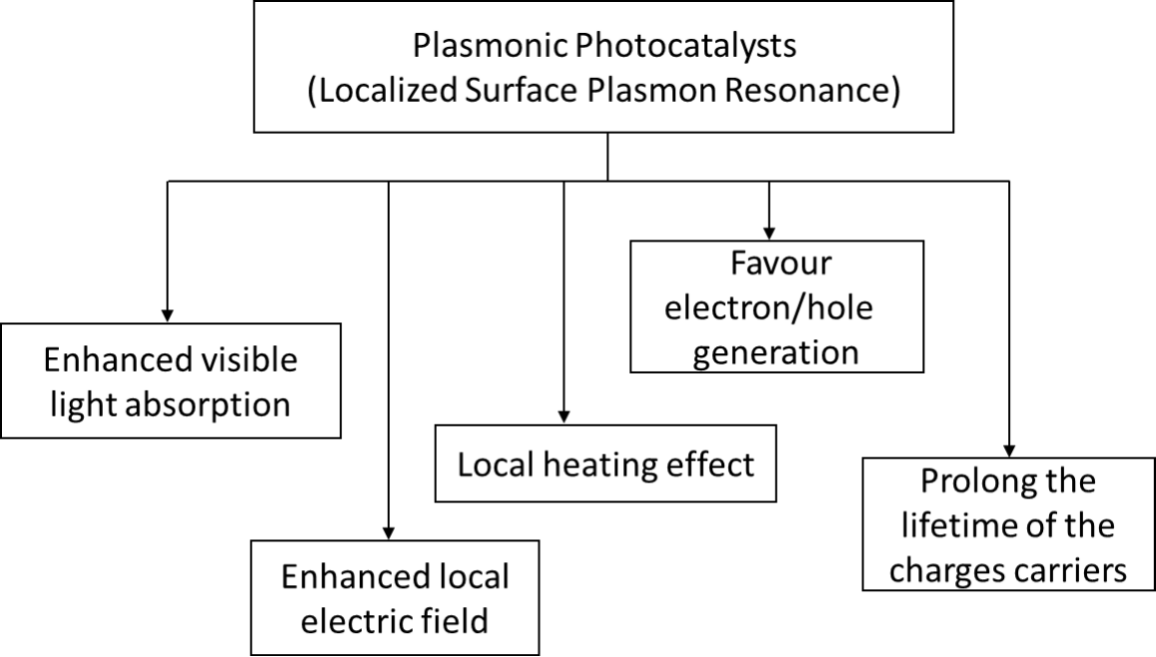
Distinctive features of plasmonics contributing to improved photocatalyst performance.

An amalgamation of noble and semiconductor metal forms an exclusive “plasmonic photocatalyst” classification. The term “plasmonic” is mainly in reference to the unique characteristics of LSPR and induced effects [6]. However, the formation of a Schottky junction does not classify as plasmonic or resonant effects. This formation is a result of notable contact between noble metal nanoparticles with a semiconductor. Plasmonic effects have been verified to increase the photocatalytic performance due to the intrinsic influences on the semiconductor photocatalyst. Plasmonic effects work to improve harvesting of visible light, prolong the lifetime of the charge carriers, improve activation of electron–hole pairs and enhance the redox reaction potential [7]. Moreover, the excitation of excess electrons and holes increases the rate of redox reaction through the heat generated. The distinct characteristic features of plasmonics are portrayed in Figure 1.

Noble metals such as Au, Ag, Pt, Pd in the form of nanoparticles are a novel candidate for high absorption of visible light owing to their strong optical absorption in the entire solar region [8–9]. The unique characteristics of the LSPR effect in noble metal allow the enhanced absorption of photon energy from the visible light spectrum. Larger metallic nanoparticles (>5 nm) produce a robust surface plasmon emission in the visible spectrum [10]. The intensity of the plasmon band is highly dependent on the morphology, surrounding medium dielectric constant, and electronic interactions between the stabilizing ligands and nanoparticles [11]. Besides, the creation of a Schottky junction with a noble metal and a semiconductor acts to retard the recombination rate of electrons and holes [12].

**Figure 2:**
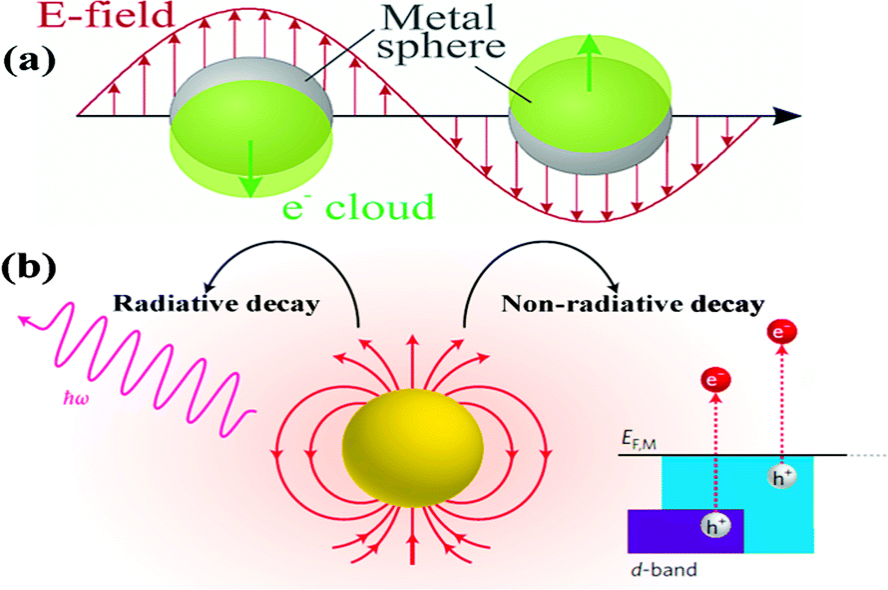
(a) Representation of localized surface plasmon resonance (LSPR) evolution in a noble metal particle irradiated by a light source. Reprinted with permission from [15], copyright 2003 American Chemical Society. (b) LSPR decay processes. Reprinted with permission from [16], copyright 2014 Nature Publishing Group.

LSPR takes place when noble metal NPs are excited by the oscillating electric field of the light. The photon frequency is designed to match with the natural frequency of the noble metal throughout the oscillation. This reduces the field on one side of the electron while increasing it on the other side of the noble metal. The development of this nonequilibrium condition results in the rearrangement of the charge density and builds an opposing electric field within the noble metal NPs. This further leads to the establishment of a coulombic restoring force and the noble metal NP electrons then experience harmonic oscillation [1,5,13–14]. These oscillating charges trigger the LSPR effects when the excited surface electric field frequency is sufficient, and the noble metal resonance leads to dynamic utilisation of visible light. A schematic representation of the phenomenon is illustrated in Figure 2. Hence, it is clear that absorption of photons emitted by the visible spectrum was promoted through LSPR and is very well ascribed to the electric polarization effect. Moreover, LSPR also speeds up the electron movement from the photoexcited noble metal to the semiconductor [5].

**Figure 3:**
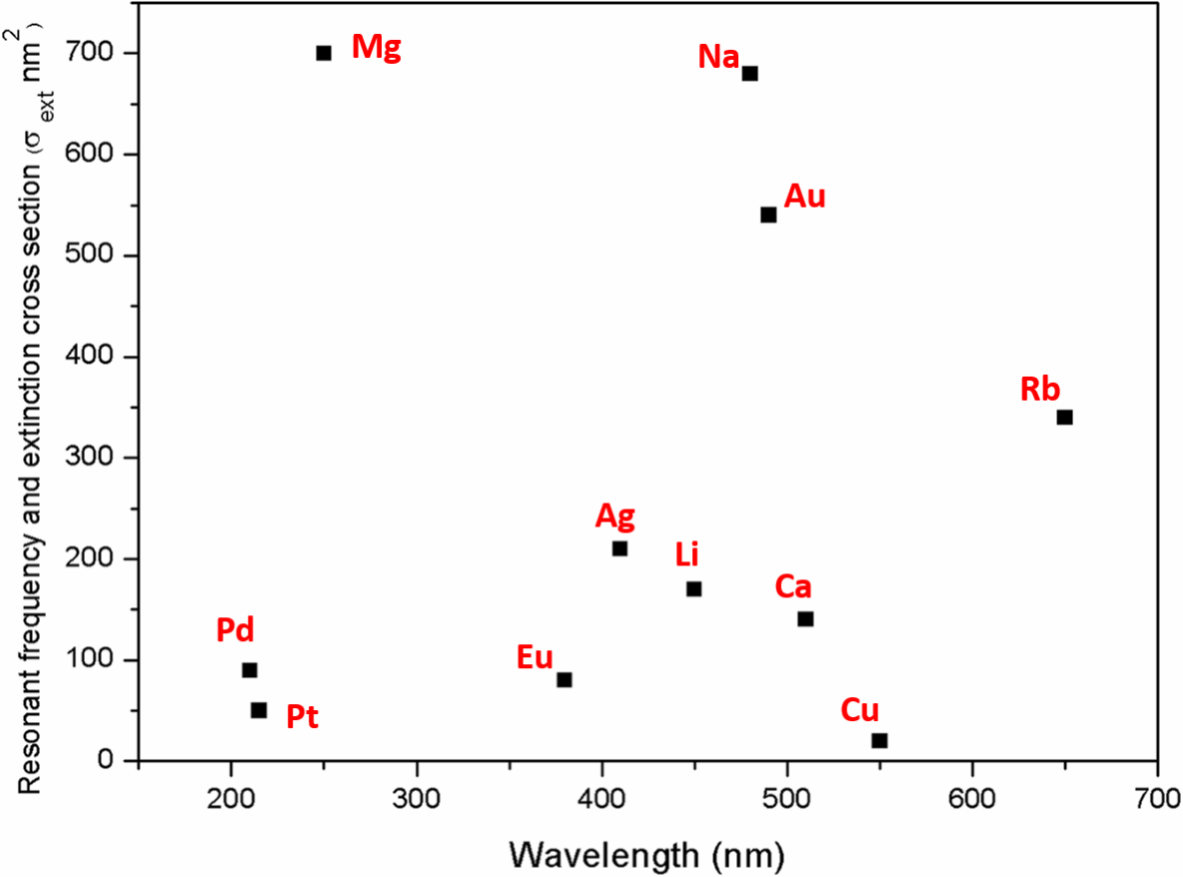
The metallic equivalent resonant wavelength for 10 nm diameter nanoparticles. Reprinted with permission from [27], copyright 2011 IOP Publishing.

The morphology, interparticle interactions and the local dielectric environment of noble metal nanoparticles is significantly influenced by the LSPR frequency [17–20]. It must be noted that not all noble metal nanoparticles of 10 nm diameter are able to utilize visible light, as shown in Figure 3. Ag and Au NPs are relatively good at harvesting visible light as compared to the others. These two noble metals can generate a stable, high electron–hole pair density [21]. However, the lower cost of Ag makes it more desirable to be utilized for broader applications as compared to Au, and therefore, most studies utilize Ag as a plasmonic inducer [22–24]. Moving away from Au one can find that palladium (Pd) in the form of NPs with a diameter of <10 nm is limited to the UV spectrum [25]. Nevertheless, the larger particle size and agglomeration of palladium particles contribute to the enhanced absorption of visible light. Mohapatra and coworkers demonstrated this where they successfully improved the harvesting potential of visible light for Pd NPs with a particle size in the range of ≈80 nm [25]. Similar results were also obtained by Kwak et al. where Pd NPs particles of ≈15 nm diameter were integrated onto TiO_2_ [26].

**Figure 4:**
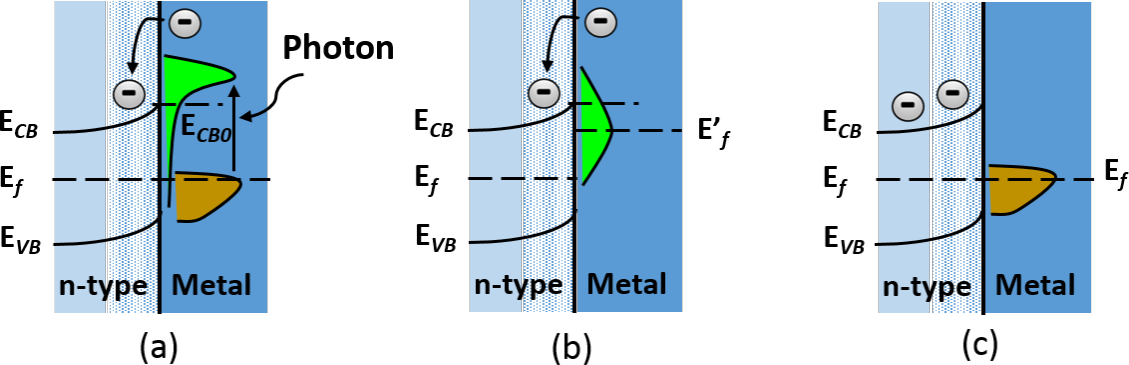
Pictorial representation of the localized surface plasmon resonance principle. Reprinted with permission from [31], copyright 2016 Springer.

**Figure 5:**
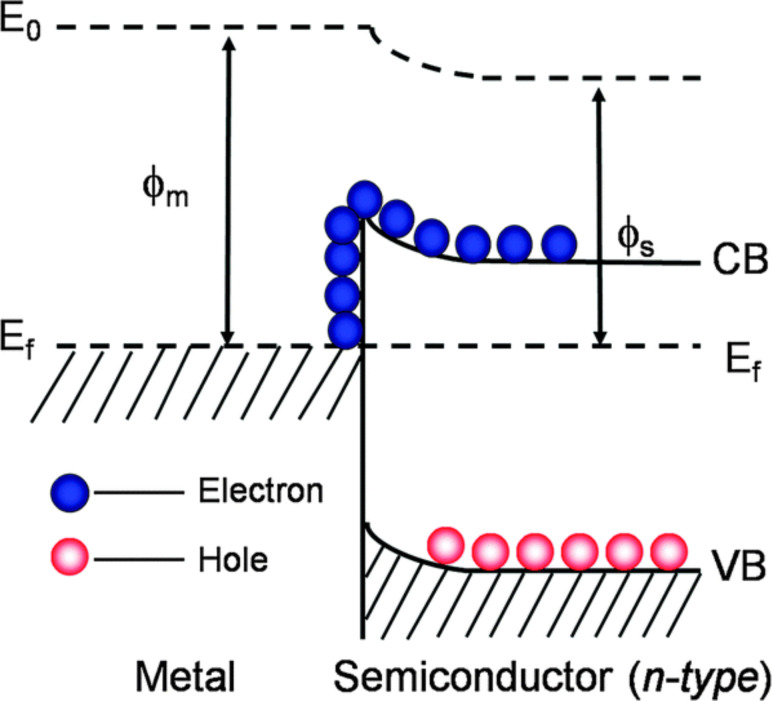
Schematic of the Schottky junction mechanism. Reprinted with permission from [35], copyright 2014 Royal Society of Chemistry.

The plasmon-assisted physico-chemical interrelation between the noble metal and semiconductor is the key for understanding electron generation and excitation. Once sufficient photon energy is obtained, it will excite the free electrons present in the noble metal to a higher Fermi level [28–29]. This movement of electrons leads to the redistribution of energy through non-equilibrium Fermi–Dirac statistics. During this redistribution, the excited electrons are transferred from the noble metal to the semiconductor and the phenomenon leads to the formation of a Schottky junction. The formed Schottky junction enables electron movement towards the semiconductor through the LSPR decay effect as illustrated in Figure 4 and Figure 5 [30], leaving behind positively charged holes at the valence band or their transfer to the counter electrode preventing recombination [16,31–35]. Figure 4a demonstrates the excited electron mobility from the thermal equilibrium to the upper energy state. Meanwhile Figure 4b illustrates the redistribution of the Fermi–Dirac distribution in a metal nanoparticle achieved through the collision. Figure 4c demonstrates the movement of excited electrons to the ordinary distribution and different regions [3,6].

Moreover, noble metals offer a rapid path for charge movement through the interface as electron–hole trap centres and allow more energetic spots for photoreactions to occur. Thus the combined traits act to readily suppress the electron–hole recombination.

### Synthesis routes for plasmonic photocatalysts

There are quite a number of ways to synthesize plasmonic photocatalysts. The most important step in their fabrication is the incorporation or deposition of noble metals onto the surface of a semiconductor. The most commonly studied semiconductor is TiO_2_, which exhibits superior photocatalysis efficiency.

Such materials have already been applied in various environmental and energy conversion applications [36]. Recently, the evolution of a metal-free semiconductor, graphitic carbon nitride (g-C_3_N_4_), has been discovered as an alternative for plasmonic photocatalysts. This metal-free semiconductor by itself has the ability to extend the absorption of visible light [37–44]. Moreover, future studies on other semiconductors such as metal chalcogenides and metal phosphides could lead to further developments for plasmonic photocatalysts to address current environmental and energy issues [45–49].

Synthesis routes such as sol–gel, hydrothermal, microwave hydrothermal, impregnation, electrochemical deposition, chemical deposition, deposition-precipitation, UV photodeposition and direct sunlight photodeposition have been reported [23,50–86]. The conventional and most frequently used methods are the sol–gel and deposition–precipitation methods. These are mostly widely preferred due to the relatively facile and direct synthesis for obtaining the target composite. However, the deposition concentration of noble metal is moderately low [3]. Meanwhile, the hydrothermal method often suffers from major metal nanoparticle aggregation issues [3]. Similarly, chemical methods result in aggregation of nanoparticles throughout the adsorption stage and thus decrease the efficiency during photocatalysis [3].

**Figure 6:**
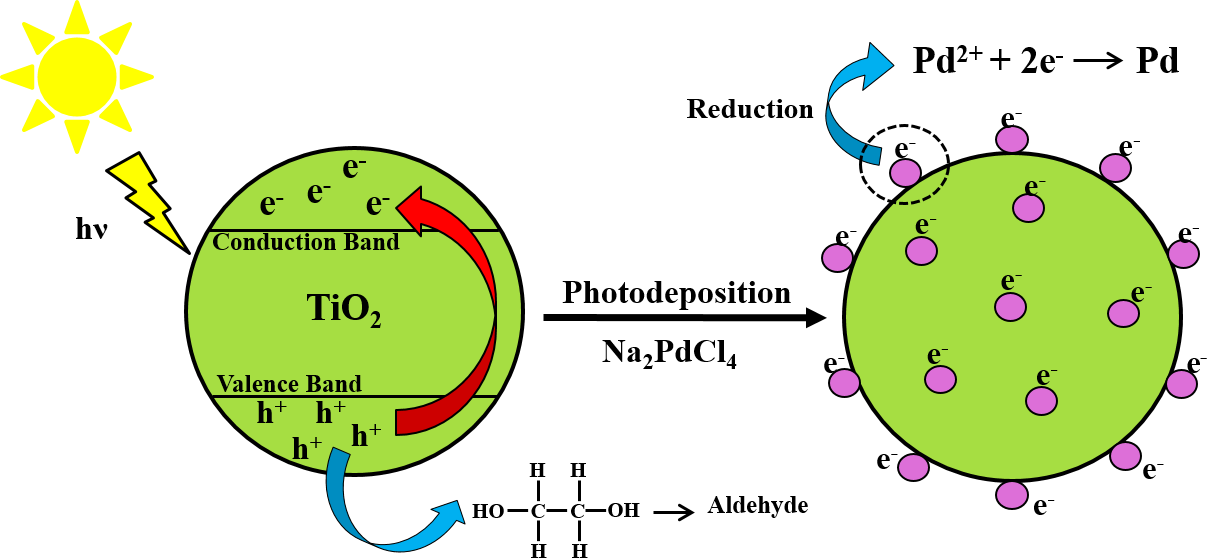
Synthesis of Pd/TiO_2_ photocatalyst via sunlight-assisted photodeposition [50].

The photodeposition method has overcome these disadvantages and produces a higher concentration of incorporated noble metal into the semiconductor composite [1]. This method is assisted by light whereby the deposition of noble metal nanoparticles takes place on the semiconductor surface. The most common light source used is a UV lamp producing a high-energy spectrum. Since most contemporary research promotes sustainable and green synthesis routes, replacing the UV lamp as the source of photons with renewable energy could be an alternative. Moreover, researchers have proven the possibility of using direct sunlight as an alternative to artificial UV light for such noble metal deposition. In a study by Leong and co-workers, they successfully achieved the deposition of Ag and Pd onto the surface of TiO_2_ via this renewable route. They also used sunlight to synthesize the LPSR-induced photocatalyst through the electron formation and mobility mechanism. Thus, the fabricated photocatalyst exhibited pronounced efficiency in generating reactive oxygen species [50–51]. In general, this specific deposition mechanism functions similar to that of TiO_2_ which has a distinctive electronic configuration structure (filled valence band and an empty conduction band). When exposed to direct sunlight irradiation, the UV light breaks the band gap energy of TiO_2_ (3.2 eV) and then activates the electrons in the valence band. Thus, activated electrons move to the conduction band, leaving behind enriched holes in the valance band. The free electrons in the conduction band are then free to react with specific precursors through the support of reducing agents, resulting uniform metallic nanoparticles deposited on the surface. The holes then react with ethylene glycol to form aldehyde. Table 1 comprehensively presents the various synthesis routes for fabrication of LPSR-induced photocatalysts. Interestingly, the fabrication of plasmonic photocatalysts using sustainable approaches showed better performance as compared to the conventional synthesis methods. The mechanism of nanoparticle formation and deposition using an sustainable approach is illustrated in Figure 6.

**Table 1:** Summary of various synthesis routes for preparation of plasmonic photocatalysts.

Plasmonic photocatalyst	Preparation method	Light source	Photocatalysis performance	Ref.

Ag-TiO_2_	UV photodeposition	visible light (457 nm) and sunlight	enhanced H_2_ production of 90 µmol (artificial visible light) and 105 µmol (sunlight) as compared to TiO_2_ (70 µmol, artificial visible light and 80 µmol, sunlight)	[52]
Ag-TiO_2_	wet chemical	sunlight	increase in Ag NP loading increased the photocatalysis efficiency: 97% degradation for 10 μM MB in 60 min and 93% for 5 μΜ MO in 40 min	[53]
Ag-TiO_2_	ultrasound	visible light (400–700 nm)	20 wt % Ag-TiO_2_ showed acetone degradation rate of 0.57 mg m^−3^ min^−1^ as compared to almost 0 mg m^−3^ min^−1^ for TiO_2_	[54]
Ag-TiO_2_	sol–gel process	visible light (18 W fluorescence TL-D tube light)	complete removal of indigo carmine (2.5 × 10^−5^ M) was achieved in 3 h	[55]
Ag-TiO_2_	hydrothermal	visible light (400–500 nm)	complete removal of rhodamine B (2 mg/L) in 180 min by Ag-TiO_2_ arrays; 55% for TiO_2_	[56]
Ag-TiO_2_	electrochemical deposition	visible light (>420 nm)	photocatalysis of Ag-TiO_2_ achieved about 80% removal of methyl blue (2 × 10^−5^ M) in 2.5 h against TiO_2_	[57]
Ag-TiO_2_	electrodeposition	visible light (400–700 nm)	complete decomposition of methyl blue with Ag/TiO_2_ core–shell nanowires within 40 min and 10 min for UV and visible light, respectively	[58]
Ag-TiO_2_	sulfydryl-assisted	visible light (>400 nm)	almost complete degradation (98%) of methyl orange was achieved by Ag/TiO_2_ as compare to TiO_2_ (30%)	[59]
Ag-TiO_2_	photodeposition	visible light (200 W halogen lamp)	TiO_2_ with 2% Ag obtained improved photodegradation of rhodamine B (10^−5^ M) with ≈30% improvement under visible light irradiation	[60]
Ag-TiO_2_	photoreduction by artificial UV light	visible light (>400 nm)	complete degradation of rhodamine B was achieved for TiO_2_ with 1 wt % Ag with initial dye concentration of 10 mg L^−1^ in 30 min	[61]
Ag-C_3_N_4_	reflux treatment	visible light (>420 nm)	enhanced photocurrent intensity (a factor of 4), photodegradation of methylene blue (by 1.8 times) and hydrogen production (by 30 times) as compared with C_3_N_4_	[62]
Ag-TiO_2_	photoreduction by direct sunlight	visible light (>420 nm)	0.3 wt % Ag/TiO_2_ showed clear photodegradation of amoxicillin and 2,4-dichlorophenol in contrast to TiO_2_	[51]
Ag-TiO_2_	photochemical reduction under Xe lamp	visible light (>400 nm)	incorporation of Ag onto TiO_2_ resulted in significant photodegradation of rhodamine B as compared to TiO_2_ (by a factor of more than 2)	[63]
Au-TiO_2_	impregnation	visible light (460–700 nm)	complete decolourization of methylene blue (1.0 × 10^−5^ M) in a short duration (10 min); complete degradation was reported for rhodamine B (2 min) and ≈25% degradation of 4-chlorophenol in 180 min	[64]
Au-TiO_2_	impregnation	visible light (LED green light)	significant enhancement was achieved with complete conversion of formaldehyde of 83.3% under visible light at 44% relative humidity	[65]
Au-TiO_2_	sol–gel	UV light (200 W Hg lamp)	Au deposition over TiO_2_ enhanced the conversion of CO_2_ to CO with a rate of 4144 µmol g^−1^ h^−1^ which is 345-fold higher than pure TiO_2_	[66]
Au-TiO_2_	deposition–precipitation	visible light (>420 nm)	the presence of Au enhanced the photocatalytic activity of both methylene orange removal and hydrogen production	[67]
Au-TiO_2_	deposition–precipitation	visible light (400–700 nm)	improved charge separation and transfer resulted to an enhanced H_2_ evolution rate	[68]
Au-TiO_2_	deposition–precipitation	sunlight	highest degradation efficiency of 97% in 50 min was reported for Safranin O dye after the incorporation of Au nanoparticles	[69]
Au-TiO_2_	deposition–precipitation	simulated solar light (Abet Technologies, Sun 2000)	H_2_ production increased up to 5–6 mmol (g^−1^h^−1^) under simulated solar irradiation and further increased with increased Au concentration	[70]
Au-TiO_2_	deposition–precipitation	sunlight	mesoporous Au/TiO_2_ with 4 wt % Au resulted in 99% removal of alizarin as compared to P25 (65%) in 80 min	[71]
Au-TiO_2_	chemical precipitation method	visible light (>305 nm)	the surface deposition approach significantly improved the photoactivity by 5–10-fold for the studied micropollutant	[72]
Au-TiO_2_	microwave–chemical reduction	visible light (>420 nm)	H_2_ production rate for Au/TiO_2_ reached 4.3 µmol cm^−2^ h^−1^ as compared to 0.47 µmol cm^−2^ h^−1^ for TiO_2_	[73]
Pd-TiO_2_	impregnation	UV and visible light (400–700 nm)	Pd activates SPR which escalates hydrogen production (by a factor of 4); the reaction requires the presence of both UV and visible light to achieve 800 µmol/g	[74]
Pd-TiO_2_	sol–gel	UV light (8 W UV lamp)	Pd-doped TiO_2_ enhanced the degradation of NO*_x_* (88%) and CO (74%) as compared to un-doped TiO_2_ (59% and 56%)	[75]
Pd-TiO_2_	hydrothermal	visible light (>400 nm)	optimal photocatalytic performance of p-nitrophenol reduction was achieved by loading 1.0 mol % Pd onto titania nanotubes; a rate constant of 0.7072 min^−1^ was reported for the photocatalytic oxidation	[76]
Pd-TiO_2_	glucose reduction	UV light (150 W Hg lamp)	Pd NPs on the TiO_2_ surface substantially increase the electron movement and act as vital sites for adsorption to promote CO_2_ hydrogenation; as a result, 1.0 wt % Pd loading yielded 355.62, 46.35, and 39.69 µmol/g for CH_4_, CO and C_2_H_6_, respectively	[77]
Pd-TiO_2_	reduction	UV light (100 W UV lamp)	photocatalytic activity of TiO_2_ impregnated with 1 wt % Pd performed well compared to TiO_2_; almost 90% conversion of n-hexane, n-octane, cyclohexane and isooctane achieved within 27, 28, 34 and 36 s, respectively	[78]
Pd-TiO_2_	chemical reduction	solar stimulator (50 mW cm^−2^, 300 W Xe lamp)	immense improvement in photocatalytic activity with enhanced H_2_ production as compared to TiO_2_; TiO_2_ decorated with 0.18 wt % Pd NPs showed an H_2_ production rate of 3096 µmol g^−1^h^−1^	[79]
Pd-TiO_2_	chemical photodeposition	solar simulator	higher decolourization (32%) for rhodamine B	[80]
Pd-TiO_2_	solar deposition	sunlight	complete degradation (97.5%) of amoxicillin was obtained within 5 h by optimum loading of 0.5 wt % Pd onto the surface of TiO_2_	[50]
Pt-loaded g-C_3_N_4_	polyol	15 W energy saving daylight	2 wt % Pt on g-C_3_N_4_ showed highest CH_4_ yield of 13.02 µmol g^−1^ as compared to unloaded g-C_3_N_4_ (2.55 µmol g^−1^ ) after 10 h of irradiation	[81]
Pt-TiO_2_	sol–gel	18 W daylight lamp	better formaldehyde degradation (98.3%) as compared to TiO_2_ (75.2%)	[82]
Pt-TiO_2_	impregnation–reduction	visible light (>450 nm)	improved catalytic performance of aniline oxidation was achieved for 2 wt % Pt particles with 12 h of photoreaction	[83]
Pt-TiO_2_	impregnation–reduction	UV light (350 W high-pressure Hg)	optimal Pt loading of 1.2 wt % exhibited increased (125-fold) H_2_ production rate compared to unmodified TiO_2_ microspheres	[84]
Pt-TiO_2_	chemical deposition	visible light (>420 nm)	rate constant of Pt-TiO_2_ for 10 mg/L nitrobenzene degradation was 2× larger than with TiO_2_	[85]
Pt-TiO_2_	UV-assisted photodeposition	visible light (>420 nm)	3 mM H_2_PtCl_6_ yielded highest photodegradation (84.27%) for methyl orange	[86]
AgCl-CN	deposition–precipitation	15 W energy saving daylight	2.5-fold increase in methane yield of was achieved for the AgCl-CN compared to CN	[87]

### Interaction of noble metals with semiconductor materials

Most of the literature claims that the incorporation of plasmonic nanoparticles with semiconductors can extend light absorption towards the visible and near infrared (NIR) spectrum. But in order to show enhanced plasmonic photocatalysis performance, the understanding of the material system plays a vital role. This includes the type of noble metal and semiconductor photocatalyst together with their morphology, porosity, crystallinity, contact form, etc. Although many semiconductor metal oxides such as N-doped TiO_2_ [88], Fe_2_O_3_ [89], CdS [90] and Bi_2_O_3_ [91–92] have been reported, this section will mainly focus on TiO_2_, which is the most widely studied. Au or Ag nanoparticles can be employed to produce an outstanding plasmonic effect in the visible and UV range [93–96]. Additionally, other noble metals such as Pt and Pd also exhibited a similar photocatalytic performance [97–98].

#### Ag, Au, Pt and Pd nanoparticles

**Figure 7:**
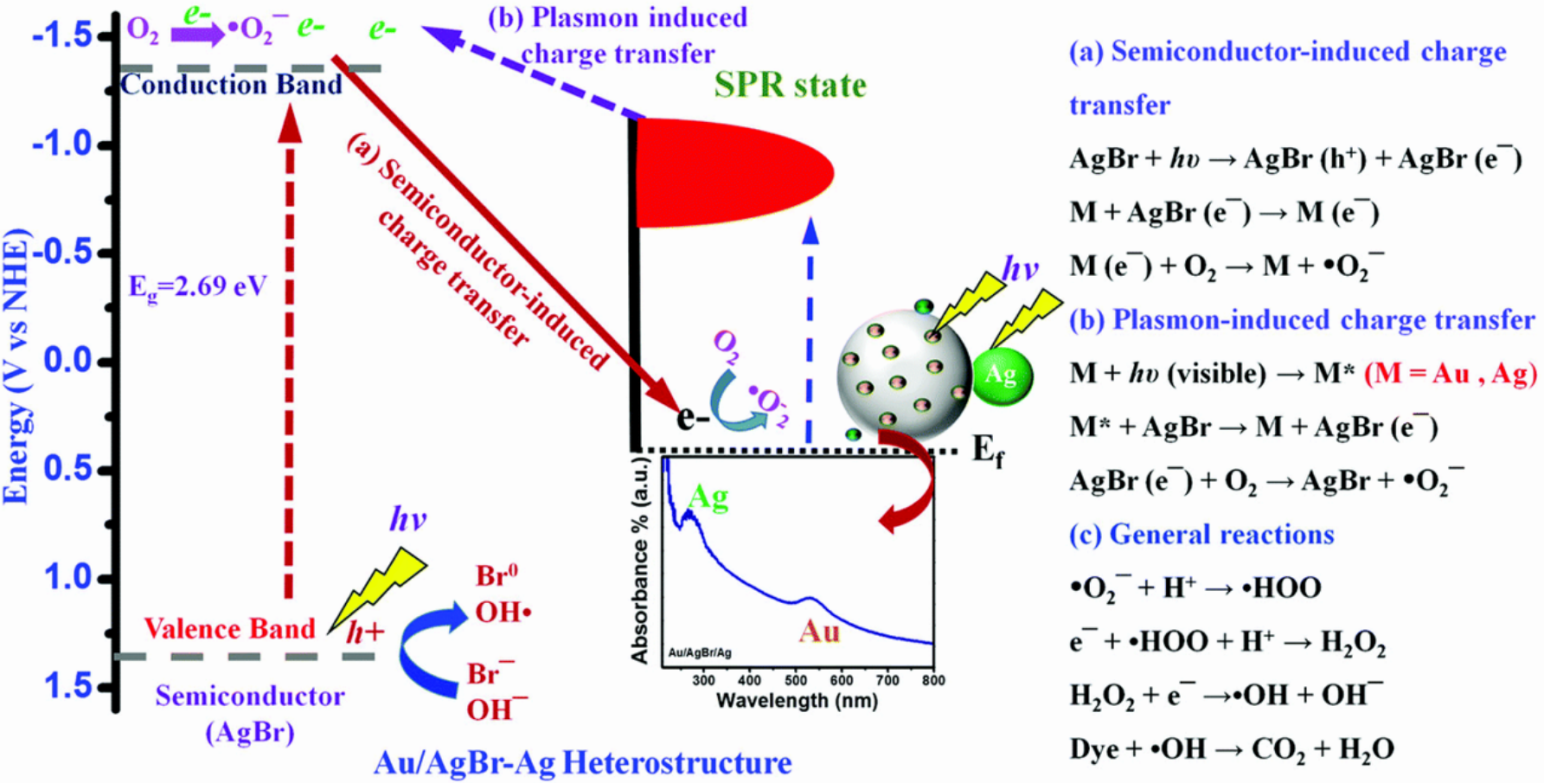
Schematic of Au/AgBr-Ag heterostructure mechanism for improved photocatalytic performance. (a) Semiconductor-excited (AgBr) electron transfer to metal (Au or Ag) NPs. (b) Plasmon-excited electron (Au or Ag) transfer to semiconductor (AgBr) NPs (e^−^ = electron, h^+^ = hole, *E*_f_ = Fermi energy, M = Au or Ag). (c) General reaction involved in mechanism of Au/AgBr-Ag heterostructure. Reprinted with permission from [103], copyright 2017 Royal Society of Chemistry.

Ag and Au are the most preferred noble metals for interaction with various types of semiconductors due to the strong LSPR produced; however, this phenomenon is mainly affected by morphology, size, and composition of the NPs as well as the dielectric properties of the surrounding medium [99–100]. Xu et al. reported that the content of metallic Ag and the extent of metallic Ag dispersion were factors which can be controlled to improve photocatalytic efficiency [101]. The LSPR of Ag in a Ag/AgCl composite was found to enhance the local inner electromagnetic field and prolong the lifetime of the charge carriers. Wang et al. observed that Ag@AgBr exhibited enhanced photocatalytic activity as compared to Ag/AgCl by a factor of 1.5. This achievement was attributed to the lower electron affinity of Br^−^ compared to Cl^−^. The variation in shape and diameter of the Ag NPs resulted in an increased frequency range of plasmonic oscillation and thus Ag@AgBr was found to readily absorb a wide range of the visible spectrum [102]. Purbia et al. incorporated Au as a secondary noble metal in a Ag@AgBr heterostructure in which the LSPR of Au NPs remained in the visible region. The resulting bimetallic (Ag–Au) coupling boosted the photocatalytic efficiency by 16-fold as compared to mono-metallic (AgBr). The increase of the resonance-excited hot-electron density on the surface of Ag and Au NPs escalated the Fermi energy level of Ag and Au, enabling the electrons to be easily injected to the conduction band of AgBr. Simultaneously, the difference in work function between the bimetallic and AgBr formed a Schottky junction to facilitate the electron transfer until a Fermi equilibrium was achieved. The schematic of the mechanism of the bimetallic Au/AgBr-Ag heterostructure and the reactive oxygen species (ROS) formation reaction as reported by Purbia et al. is depicted in Figure 7 [103].

**Figure 8:**
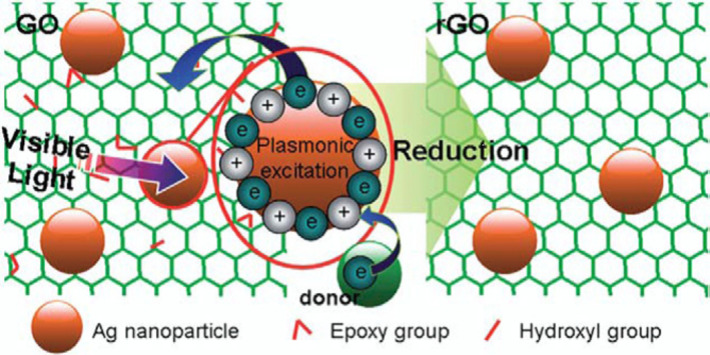
Photodegradation of GO in the presence of an electron donor (Ag NPs). Reprinted with permission from [95], copyright 2011 Royal Society of Chemistry.

Another such similar finding was reported on the photoreduction of graphene oxide (GO) to graphene or reduced graphene oxide (rGO) by Wu et al. Their study revealed the photocatalytic Ag NP reduction at λ > 390 nm [95]. The schematic diagram representing the interaction of GO with Ag is shown in Figure 8. The LSPR effect on the Ag NPs generated a strong oscillating local electric field that enhanced the excitation of metallic charge carriers. The subsequently excited electrons were transferred to the conduction band of GO, yielding GO reduction and oxidation of Ag NPs [6].

Sun et al*.* described the role of Ag in the Ag@C composite. The composite displayed a broad LSPR absorption band at ≈630 nm [93]. The Ag@C nanocomposite exhibited excellent removal of aqueous rhodamine B and gaseous acetaldehyde (CH_3_CHO) under visible light irradiation. There have been some research reports on the combination of either Pd or Pt (considered to induce a reduced plasmonic effect) with Au or Ag, (considered to induce an enhanced plasmonic effect) in which the former acts as an electron sinker for more efficient electron–hole pair separation [6]. For instance, Shuang et al. studied one such combination where they decorated TiO_2_ nanopillar arrays with both Au and Pt NPs and achieved a photocatalysis efficiency of 21 and 13 times higher than for pure TiO_2_. Their excellent results were attributed to the synergistic effect of Pt NPs which act as an electron trapper and the SPR of Au NPs [104].

#### Interactive plasmonic photocatalytic systems

**Figure 9:**
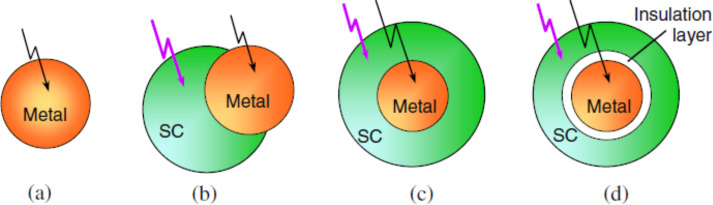
(a) Pure metal nanoparticles (NPs) without any semiconductor. (b) Metal NPs partially embedded into the semiconductor and partially exposed to the environment. (c) Metal NPs having a direct electrical contact by being fully embedded within the semiconductor without being exposed to the environment. (d) Metal NPs isolated from the semiconductor by a non-conducting layer to prevent direct electric contact. Reprinted with permission from [6], copyright 2013 IOP Publishing.

The various plasmonic photocatalytic systems differ with respect to the type of interaction between the noble metal and the semiconductor. The taxonomy of the plasmonic photocatalytic systems together with their interaction patterns are schematically explained in Figure 9 [6].

Figure 9c clearly shows the embedded form of the noble metal/semiconductor system in which the metal is completely embedded into the semiconductor. This type of system benefits from the full advantages of Schottky junction formation with enhanced electron mobility as compared to partially embedded structures (Figure 9b). In addition, noble metal NPs that are embedded inside the semiconductor layer are less likely to dissolve, aggregate and detach. These advantages are helpful to increase the scattering of the NPs in the semiconductor and to confirm the stability of the photocatalysis reaction. However, noble metal NPs do not interact with the surrounding environment in this system (as do organic molecules in the solution) and thus lack the potential for redox reactions to occur. The generated charge carriers are trapped by the presence of noble metal. This leads to saturation when accumulating charge carrier reaches it limits. Thus, the gathered charges are finally trapped and allow a slow dispersion across the Schottky junction. However, in order to understand the mechanism of the LSPR effect, the Schottky junction, and the Fermi and mobilisation of electron/holes across the noble metal and semiconductor, either advanced characterisation tools or a theoretical simulation is necessary for detailed understanding of the circumstances.

#### Advanced characterization and theoretical simulation

**Figure 10:**
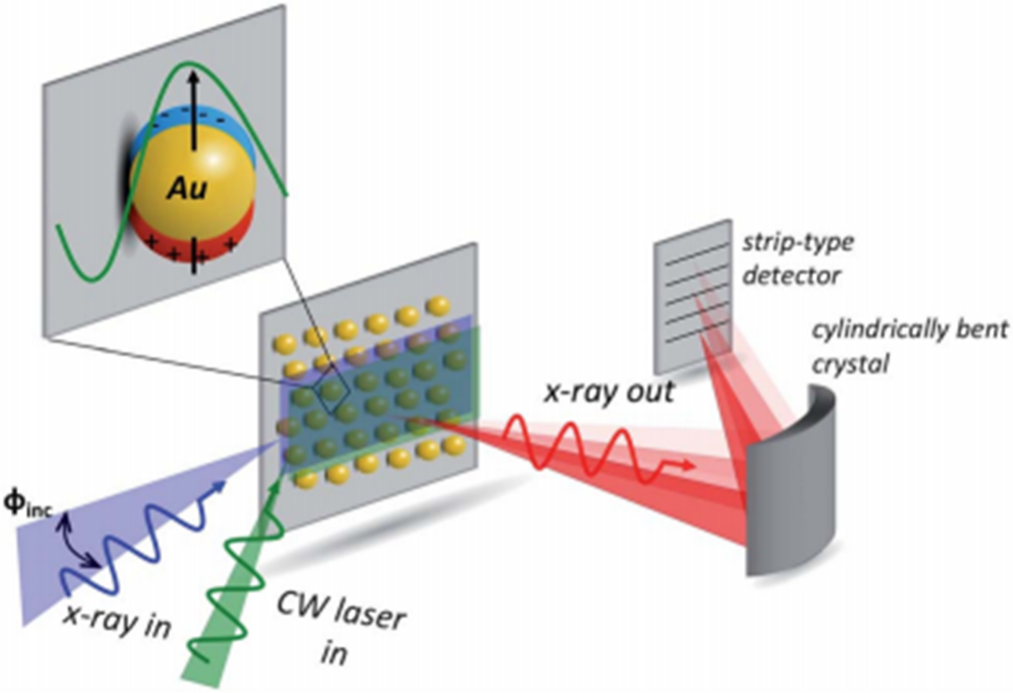
High-resolution X-ray absorption spectroscopy (HR-XAS) experiment used to determine the changes in the Au L_III_-edge induced by 100 mW continuous wave laser excitation of the localized surface plasmon at 532 nm. Reprinted with permission from [105], copyright 2013 Royal Society of Chemistry.

The electron transfer mechanisms of plasmonic/semiconductor hybrid systems have been reported elsewhere. However, the principal mechanism that governs the plasmon excitation and electron injection into the semiconductor are still unclear. The verification that plasmon-excited electrons in Au NPs possess sufficient energy to overcome the Schottky junction to be injected into TiO_2_ was confirmed using high-resolution X-ray absorption spectroscopy (HR-XAS) [105]. The adopted experimental setup is depicted in Figure 10. The significant spectral variations observed by X-ray absorption spectroscopy (XAS) and resonant inelastic X-ray scattering (RIXS) suggest that electrons injected from Au NPs upon LSPR excitation could survive longer and become trapped at a Ti site near the surface of TiO_2_ [106]. More detailed work needs to be carried out to identify the individual contributions from different plasmonic effects such as hot-electron injection, generation of electromagnetic field and plasmon-induced heating [107]. The measurement of plasmonic photoelectrodes with polarized irradiation along various axes was combined with theoretical simulations based on the finite element method (FEM). In situ XAS was used to understand the electronic structural changes caused by the electromagnetic field upon the surface of plasmonic materials [108]. Designing the physical parameters of plasmonic metal nanostructures such as particle size, work function, surface facet and plasmonic band is a challenging task that demands numerical simulation. It is known that the photocatalysis performance is affected by the noble metal particle size and thus finite difference time domain (FDTD) simulations were studied to reveal spectral and spatial features of the plasmonic field [109–110]. The FDTD simulations probe the effect of the particle size for optimizing the performance of catalytic systems. Some research groups have performed FDTD simulations to elucidate the contribution of the LSPR feature in the Cu_7_S_4_@Pd catalyst. They found that Pd NPs showed weak LSPR absorption at 808, 980 and 1500 nm, while Cu_7_S_4_ exhibited obvious electrical field enhancement at these wavelengths; thus Cu_7_S_4_ was found to be the dominant contributor to the LSPR feature [111]. Using a similar simulation method, it was claimed that the enhanced photo-electrochemical water splitting performance of Pd@BiVO_4_ was attributed to the hot-electron injection from Pd NPs upon SPR excitation in the vis–NIR region [112].

### Identification of reactive radicals

**Figure 11:**
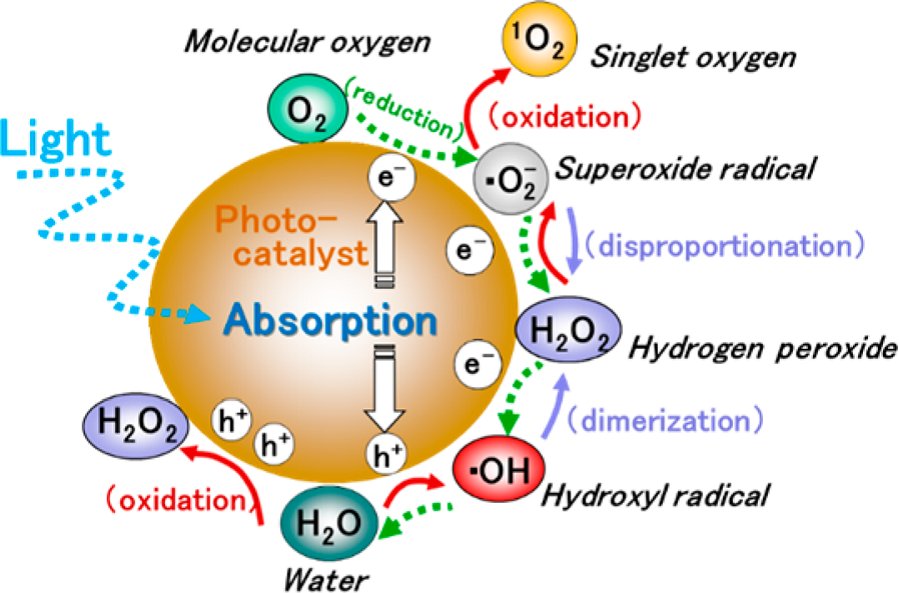
Generation of reactive oxygen species (ROSs) in the photocatalytic reduction and oxidation of O_2_ and H_2_O. Reprinted with permission from [115], copyright 2017 American Chemical Society.

It is quite obvious that photocatalysis is supported by redox reactions caused by photo-induced formation of electrons (e^−^) and holes (h^+^). Several reactive species are produced on the heterogeneous solid surfaces of photocatalysts during the oxidative and reductive reactions in photocatalysis. Thus photocatalysis can be practically employed with water vapour under aerobic conditions, whereby photocatalysis involving oxygen (O_2_) and water (H_2_O) as reaction species is vital. The species to which oxygen converts with high reactivity are generally called reactive oxygen species (ROSs) and four such major ROSs are recognized, namely hydroxyl radical (•OH), superoxide anion radical (•O_2_^−^), singlet oxygen (^1^O_2_) and hydrogen peroxide (H_2_O_2_). Since the redox reaction takes place during the photocatalysis reaction, ROSs are produced sequentially both from O_2_ and H_2_O as illustrated in Figure 11 [113–115]. In general ROSs of •OH, H_2_O_2_, •O^2−^ and ^1^O^2^ would be generated in this order by the stepwise oxidation of H_2_O. On the other hand, the stepwise reduction of O_2_ generates •O^2−^, H_2_O_2_ and •OH.

The detection or quantification of these ROSs in the presence of a reactant pollutant is crucial for practical applications and such quantification can be correlated to the quantum efficiency (i.e., formation of ROSs for incident photon). Direct phosphorescence detection during the photocatalytic reaction would be beneficial for detection of ^1^O_2_. For •OH, probing in solution could be observed from the surface reaction by perceiving the location of the probe molecules. In some cases the detection of •O_2_^−^ and H_2_O_2_ after the photocatalytic reaction could be also possible. This review covers various studied detection methods used to identify the active radicals (quantum analysis) involved in the photocatalysis along with the uncertainties involved. Most of the reports of the present review concern ROSs in aqueous suspensions of photocatalyst powders. However, in some cases, reactions under gaseous conditions, whose behaviour might be different from those in aqueous suspensions, were involved.

### Quantification methods for H_2_O_2_

Although the generated ROSs subsequently decay, H_2_O_2_ is the most stable of the molecules and can be detected separately. As of now, various methods have been employed for its quantification in photocatalysis reactions and could be classified as (1) direct optical absorption in UV and IR regions, (2) coloration and (3) fluorescence probe.

#### Direct optical absorption

**Figure 12:**
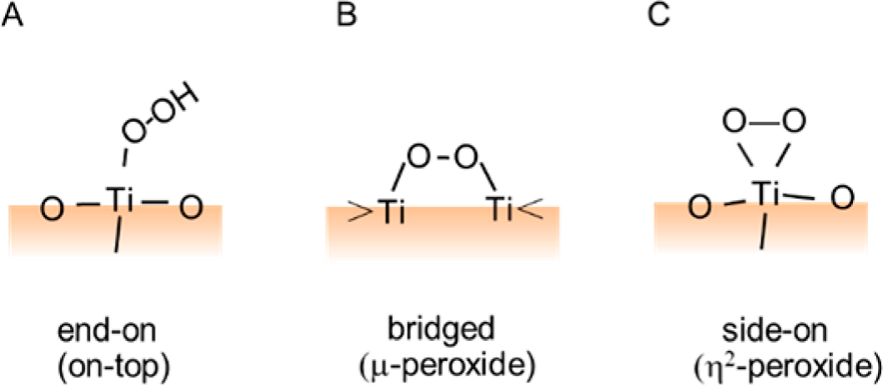
Plausible structural formation of adsorbed H_2_O_2_ on TiO_2_ surface (a) end-on (b) bridged and (c) side-on. Reprinted with permission from [115], copyright 2017 American Chemical Society.

The direct detection of H_2_O_2_ in solution by measuring UV spectra is difficult due to the weak molar absorption coefficient of H_2_O_2_ (0.01 M^−1^ cm^−1^) at 360 nm, which gradually increases up to 13 M^−1^ cm^−1^ at the wavelength of 260 nm [115]. Meanwhile, the optical absorption in the IR region can be detected by observing the peak of O–O stretching of H_2_O_2_. Figure 12 shows three plausible types of structural formation of adsorbed H_2_O_2_ on to the surface of TiO_2_. A side-on peroxide structure (Figure 12c) appeared for the absorption band on rutile at 820–940 cm^−1^ [113]. The peaks at 838 and 877 cm^−1^ were assigned to the end-on peroxide (see Figure 12a), and that at 812 cm^−1^ was assigned to the bridged peroxide (see Figure 12b) [115]. The signal observed at 928 cm^−1^ was tentatively assigned to the triangle peroxide (see Figure 12c) [115].

#### Coloration methods

Coloration is another common method adopted for the quantification of H_2_O_2._ In this method, iodide [116–117], Ti^4+^ ions [118] and *N*,*N*-dimethyl-*p*-phenylenediamine (DPD) [119–120] were used to detect H_2_O_2_ during photocatalysis. For the iodide method, the solution was mixed with KI and sodium acetate buffer containing a few drops of catalyst (ammonium dimolybdate) for the oxidation of I^−^ by H_2_O_2_. Thus, I^3−^ was obtained in the solution and was measured at 360 nm. Alternatively, when Ti^4+^ ions were used as indicators, yellow-coloured metal complexes with H_2_O_2_ were formed and examined at 410 nm. [118]. The DPD method is based on the oxidation of DPD by H_2_O_2_ catalysed with horseradish peroxidase. The radical cation DPD^+^ exhibits a fairly stable colour with an absorption peak at 551 nm [119–120].

#### Fluorescent probe

**Figure 13:**
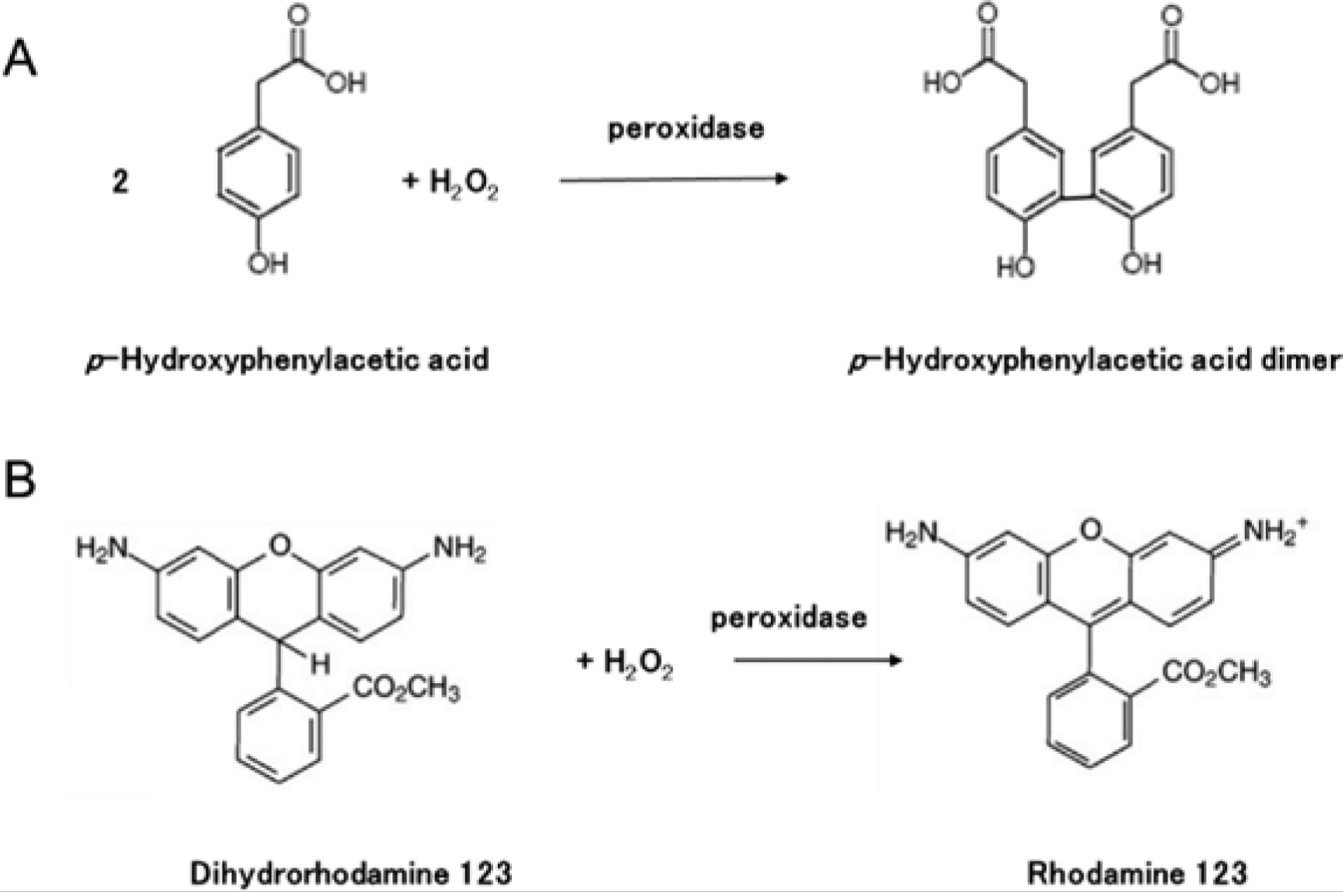
Reactions involved in the detection method of H_2_O_2_ with fluorescence probes (a) p-hydroxyphenylacetic acid (HPA) and (b) dihydrorhodamine 123. Reprinted with permission from [115], copyright 2017 American Chemical Society.

In this method a fluorescent dimer is created by reacting determining species (H_2_O_2_) with p-hydroxyphenyl- acetic acid mediated with horseradish peroxidase as a catalyst. The complete reaction scheme is explained in Figure 13a [115]. The intensity of the generated fluorescence is analysed using a fluorescence spectrophotometer with an emission wavelength of 408.5 nm excited at 316.5 nm. The concentration of the peroxide is directly proportional to the intensity of the created fluorescence as explained by the Beer–Lambert law [121–123]. Alternatively, a fluorescence probe reagent, dihydrorhodamine 123 was also reported to detect H_2_O_2_ in photocatalytic systems where dihydrorhodamine 123 is oxidized to a fluorescent molecule, rhodamine 123, by the reaction with H_2_O_2_ and peroxidase as shown in Figure 13b [115].

### Detection methods for ^1^O_2_

^1^O_2_ is singlet oxygen, which is an excited state of O_2_. It can be deactivated to the original stable O_2_ without being involved in chemical reactions or electron transfer. The detection methods of ^1^O_2_ are focussed on (1) electron magnetic resonance and (2) fluorescence probe methods.

#### Electron spin resonance detection with probe reagents

**Figure 14:**
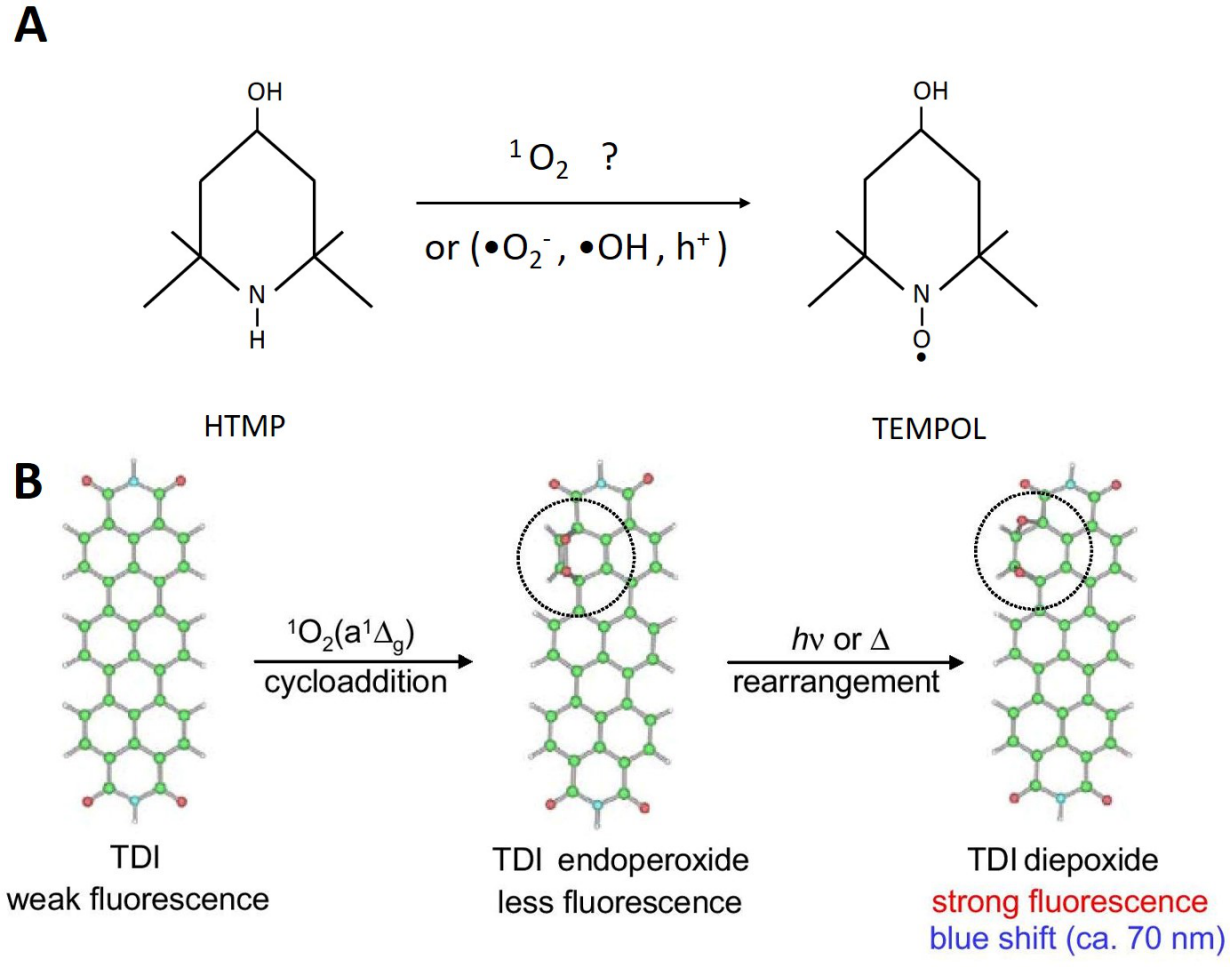
(a) Reaction of HTMP to TEMPOL. Reprinted with permission from [115], copyright 2017 American Chemical Society. (b) Fluorescence detection of ^1^O_2_ with TDI. Reprinted with permission from [125], copyright 2007 Springer Science + Business Media.

For the detection of singlet oxygen in photocatalysis, 4-hydroxy-2,2,6,6-tetramethylpiperidine (HTMP), a well-known stable nitroxide radical (4-hydroxy-2,2,6,6-tetramethylpiperidine 1-oxyl, TEMPOL) is used to generate the corresponding 1-oxyl radical by the reaction with ^1^O_2_ [124]. The final product is subjected to electron spin resonance (ESR) for the quantification of ^1^O_2_ as shown in Figure 14a.

A terrylene diimide (TDI) derivative that can produce fluorescent diepoxide through cycloaddition (Figure 14b) was adopted to detect ^1^O_2_ generated in air. This TDI derivative is usually coated on a glass plate and faced the photocatalyst with an air gap through which ^1^O_2_ diffuses. The detection of single-molecule fluorescence can be observed using a light microscope [125–126].

Finally, since the ^1^O_2_ species possess paramagnetic properties caused by the orbital angular momentum, they can be quantified by direct ESR detection [127]. Therefore, an ESR spectrometer with a microwave frequency of about 9 GHz (X-band) could be used to observe the quartet signal of ^1^O_2_ in the gas phase at 950 mT. Nevertheless, this detection method has not yet been applied to detect ^1^O_2_ produced during photocatalysis.

#### Detection methods of •OH

The •OH radical is often regarded as the most effective reactant for photocatalytic decomposition. Among the various ROSs, the rate constant of •OH is almost at the diffusion limit and hence the reactivity of •OH is considerably high. The detection methods utilized for •OH in photocatalysis are focussed on (1) laser-induced fluorescence (LIF), (2) spin-trapping ESR and (3) fluorescent probe methods.

#### Laser-induced fluorescence methods

**Figure 15:**
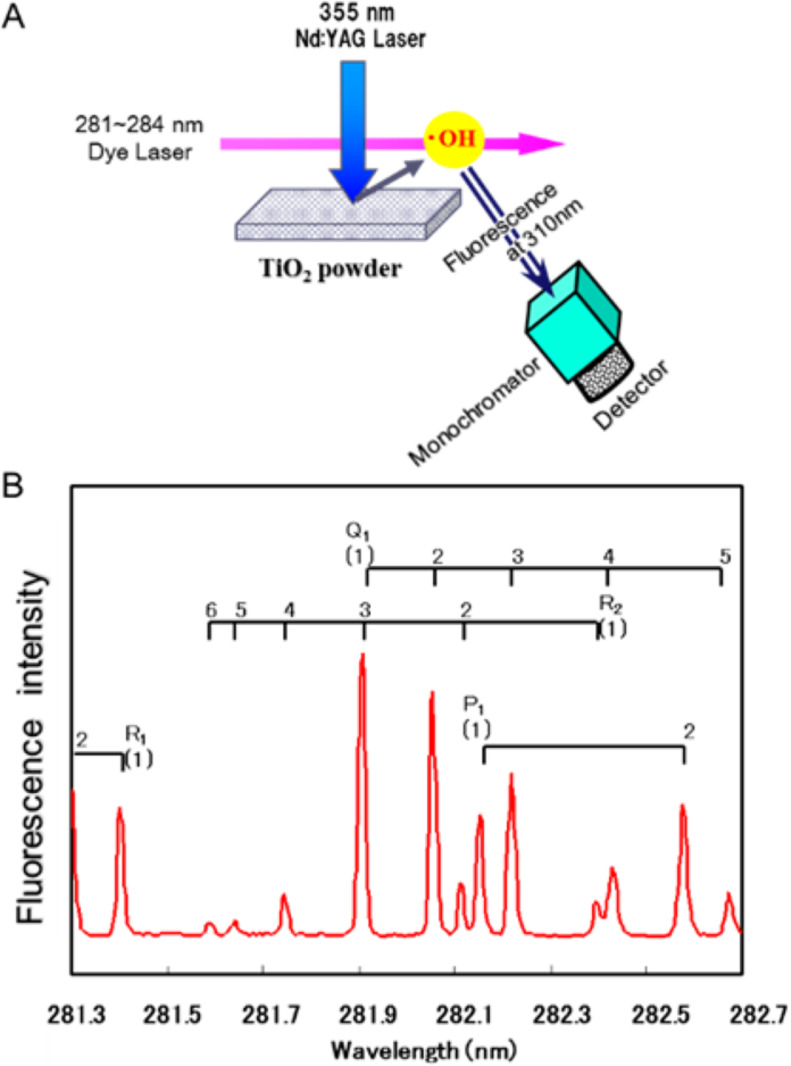
(a) Laser-induced fluorescence detection of •OH released from an irradiated TiO_2_ surface. Reprinted with permission from [128], copyright 2007 American Chemical Society. (b) Obtained excitation spectrum of •OH radicals. Reprinted with permission from [115], copyright 2017 American Chemical Society.

This highly sensitive method is employed to detect an low concentration of •OH radicals in the atmosphere. Figure 15a shows the experimental framework to measure the •OH radicals generated from irradiated TiO_2_ [128]. A dye laser, which acts as a source of the emission wavelength at 310 nm, was used to calculate the intensity of the fluorescence emitted from •OH radicals. The •OH radicals emitted from the photoexcited TiO_2_ surface to the gas phase were confirmed by the LIF spectrum (see Figure 15b) with the characteristic rotational structure of the transition energies [115].

#### Spin-trapping electron spin resonance

**Figure 16:**
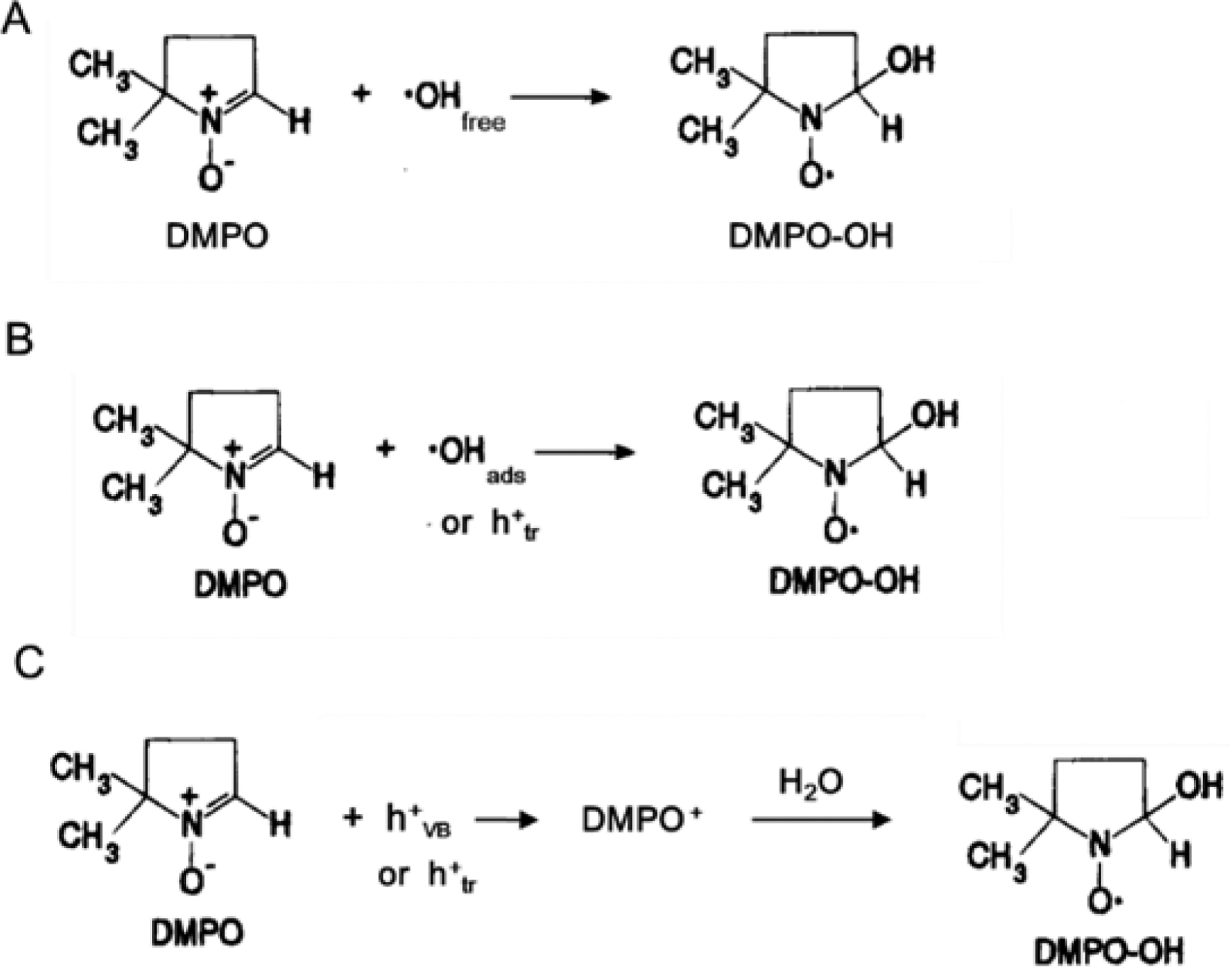
Reaction routes for detection of •OH radicals with a DMPO spin-trapping reagent. Reactions with •OH radicals (a) in solution, (b) at the surface, and (c) indirect reaction via oxidation of DMPO. Reprinted with permission from [115], copyright 2017 American Chemical Society.

Spin-trapping ESR is a conventional method and most often utilized to detect the •OH generated in biological systems with the support of a spin-trapping reagent, namely 5,5-dimethyl-1-pyrroline *N*-oxide (DMPO) [129–130]. The unstable •OH radicals released during the photocatalysis react with DMPO to convert stable DMPO–OH radicals and are detected by ESR spectroscopy. There is a high probability that the valence-band holes might alternatively oxidize the spin-trapping reagents before the formation of •OH radicals. Figure 16 shows a three-step process for DMPO–OH radical formation in photocatalysis.

#### Fluorescent probe

**Figure 17:**
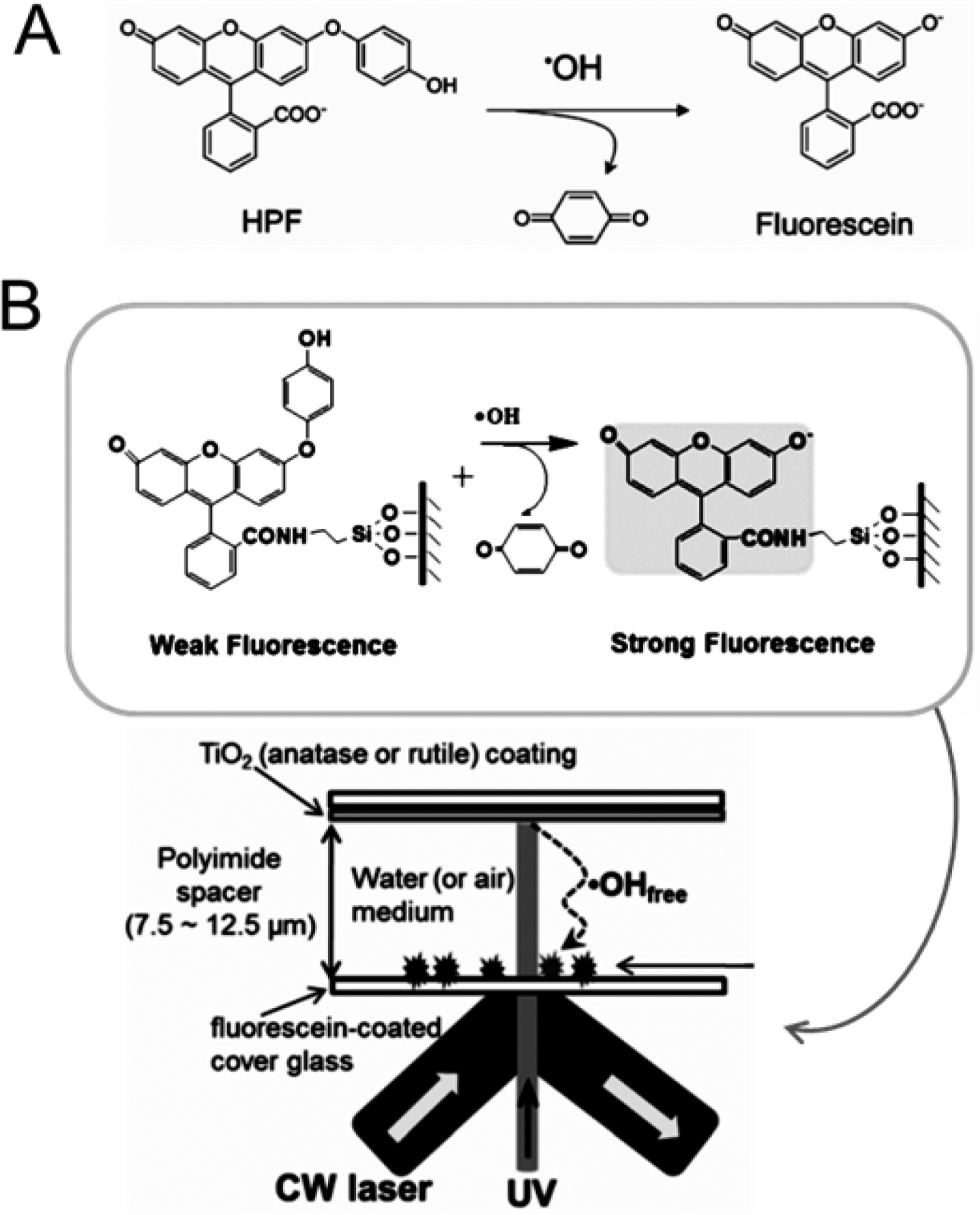
(a) Usage of fluorescence probe HPF to detect •OH radicals. (b) Experimental setup for the single-molecule detection of photogenerated •OH in H_2_O. Reprinted with permission from [131], copyright 2014 Wiley-VCH Verlag GmbH & Co.

The fluorescein, 3-(*p*-hydroxyphenyl)fluorescein (HPF) and 3-(*p*-aminophenyl)fluorescein (APF) dyes have been used as reagents in photocatalytic reactions [131]. A strongly emissive fluorescein molecule, as shown in Figure 17a, was formed when HPF selectively reacts with •OH radicals. However, it does not react with the other ROSs, such as •O_2_^−^, ^1^O_2_, and H_2_O_2_. A fluorescein-coated glass plate supported with HPF molecules is employed and a silanol group separates the TiO_2_ coating glass plate with a spacer, as shown in Figure 17b. Polyimide films were used to control the distance between the two glass plates, and the space was filled with air-saturated water. In this way, it was confirmed that the •OH generated on the photocatalysts could diffuse to the HPF-coated glass.

### Detection methods of •O_2_^−^

#### Spin-trapping electron paramagnetic resonance

**Figure 18:**
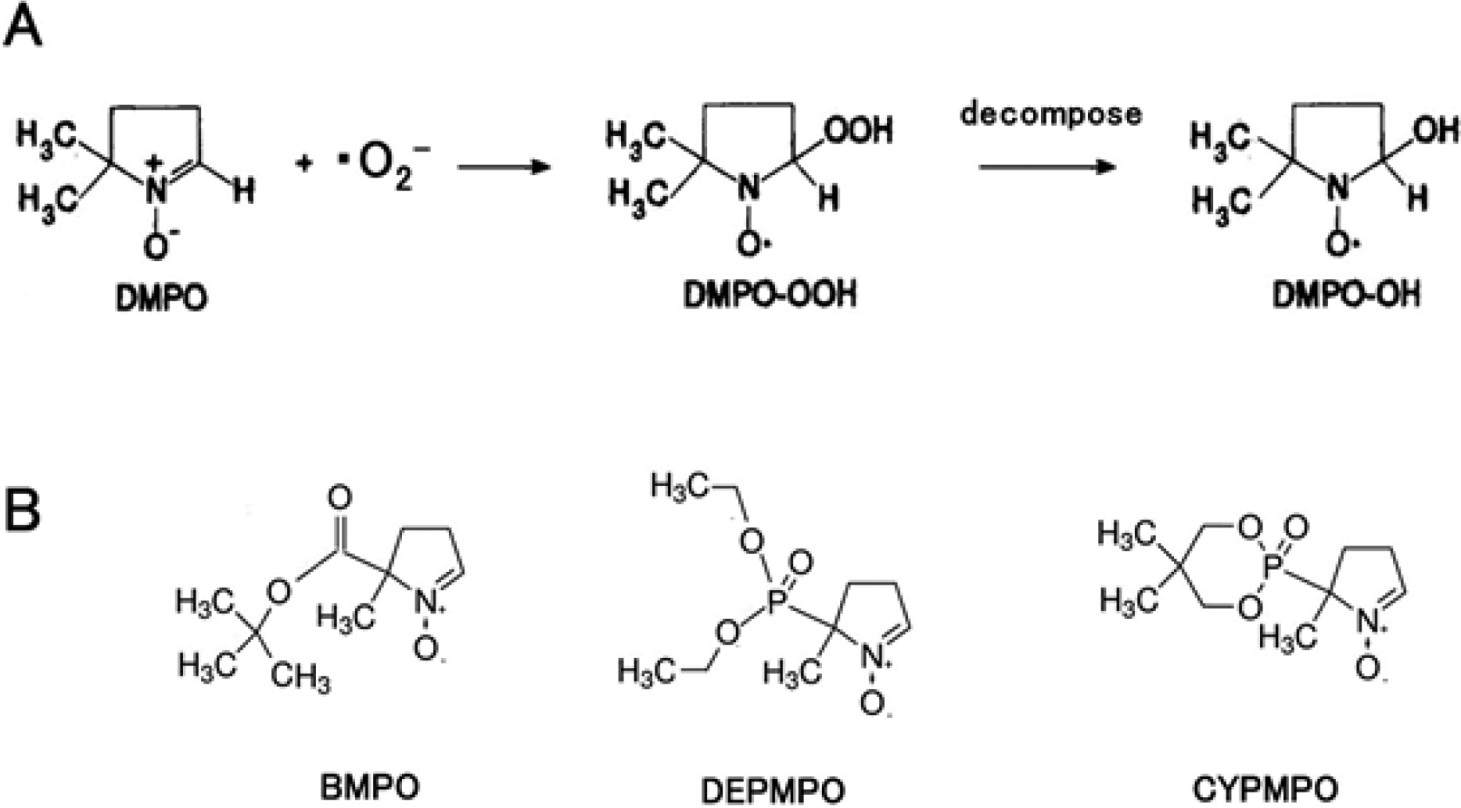
(a) Reactions involved in the detection of •O_2_^−^ with DMPO. (b) Chemical structures of the spin-trapping reagents for •O_2_^−^, BMPO, DEPMPO and CYPMPO. Reprinted with permission from [115], copyright 2017 American Chemical Society.

The detection the •O_2_^−^ in aqueous solution by EPR can be performed with the presence of DMPO as the trapping agent (Figure 18a). Unfortunately the reaction rates of DMPO with •O_2_^−^ and •O_2_H are extremely small as compared with the •OH radical. In addition, the •O_2_^−^, which is drawn to DMPO, is unstable and it converts to •OH adducts (Figure 18a).Thus, the detection of •O_2_^−^ with DMPO is not facile [115].

Spin-trapping reagents such as 5-diethoxyphosphoryl-5-methyl-1-pyrroline *N*-oxide (DEPMPO) [132] and 5-*tert*-butoxycarbonyl-5-methyl-1-pyrroline *N*-oxide (BMPO) [133] and 5-(2,2-dimethyl-1,3-propoxycyclophosphoryl)-5-methyl-1-pyrroline *N*-oxide (CYPMPO) [134] have been reported. The chemical structures of these reagents are explained in Figure 18b.

### Future perspective

Even though remarkable progress has been achieved in this area of research, some challenges are still to be met. The future perspective on the realistic application of plasmonic photocatalysts will focus on the scalability, cost and sustainability from the perspective of synthesis route to application. In addition, the theory behind the plasmonic-induced electron transfer mechanism still remains questionable and thus necessitates the advanced characterization and theoretical simulation at the electronic level.

#### Near-infrared plasmonic materials for optimized solar absorption

**Figure 19:**
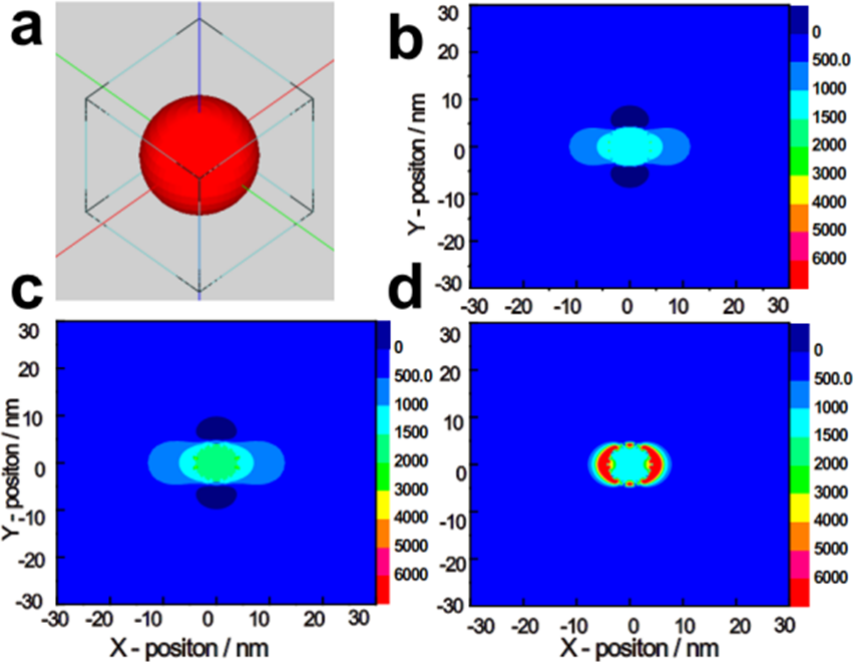
(a) FDTD simulation set up for Cu_7_S_4_. (b–d) 2D contour map of the electric field intensities around the Cu_7_S_4_ nanocrystal under illumination at 808 nm (b), 980 nm (c) and 1500 nm (d), respectively. Reprinted with permission from [111], copyright 2015 American Chemical Society.

In the preceding decades, much research was focused on the application of Au and Ag as plasmonic metals for utilising the visible region that consists of ≈43% of the solar spectrum against NIR [135]. The high carrier concentration (≈5.9 × 10^22^ cm^−3^) of Au and Ag enables their intense LSPR excitations in the visible region [136]. The resonance in both Au and Ag can be controlled, but their high resistive loss, earth rarity and high cost are all disadvantages which limit them from being produced in large scale for real-world applications [21]. It is well-known that NIR wavelengths (760–3000 nm) account for ≈54% of the solar spectrum compared to the visible, which necessitates the search for plasmonic materials capable of utilising photons across the NIR region [137]. Compared to Au and Ag, conducting oxides and semiconductor nanomaterials possess lower carrier concentrations that are useful for their resonance in the NIR range [16]. For example, when coupled with Pd, copper chalcogenide (Cu_7_S_4_) utilizes NIR range photons through LSPR, and Pd traps the hot-holes for photocatalysis reactions, including oxidation of benzyl alcohol, hydrogenation of nitrobenzene and the Suzuki coupling reaction. The strong electrical field intensity at 1500 nm (Figure 19) reveals that the LSPR is more significant when the irradiation wavelength is near to the Cu_7_S_4_ LSPR peak [111]. Likewise, tungsten oxide (WO_3–δ_) nanocrystals showed intense NIR absorption with an LSPR peak at ≈900 nm [138]. The plasmonic resonance of semiconductors could be manipulated by tuning the stoichiometric composition, dopant concentration, or phase transitions [139–140]. The manipulation of the stoichiometric ratio of semiconductors could increase the free-charge density and promote the LSPR arising from collective oscillations of excess free charges on semiconductor surface, thus enhancing the NIR absorption abilities [141–142]. Interestingly, Cu_2–_*_x_*Se nanocrystals (NCs) showed distinct NIR plasmon band due to the formation of copper vacancies in the material through exposure to oxygen or to a Ce(IV) complex [143]. Similarly, the LSPR band in stoichiometric Cu_2–_*_x_*S, Cu_2–_*_x_*Se and Cu_2–_*_x_*Te was improved by converting them into their nonstoichiometric counterparts via oxidation/reductive reactions [142]. Besides, the carrier concentration of aluminium-doped zinc oxide (AZO) can be shifted from 0.5 to 10 × 10^20^ cm^–3^ by varying the concentration of Al, thus contributing to the wide-range SPR (2200–880 nm) [144]. Despite the tunable plasmonic features of semiconductors, some plasmonic semiconductors are photocatalytically inert due to their unfavourable band edge position compared to the redox potential of targeted species. An effective approach to overcome this restriction was to integrate the nonstoichiometric materials (tungsten oxide (W_18_O_49_)) with graphitic carbon nitride (g-C_3_N_4_). The g-C_3_N_4_ was used to effectively capture the LSPR-excited hot electrons from W_18_O_49_, enabling an efficient photocatalytic reaction [145]. Similarly, TiO_2_ was coupled with W_18_O_49_ for effective photocatalysis under full solar spectrum conditions. Another advantage of such hybrid nanostructures is their ability to facilitate the spatial charge separation of photoinduced carriers by creating an alternate permutation of band edges at the interface [146]. Some researchers found that both titanium nitride (TiN) and zirconium nitride (ZrN) displayed similar optical properties as Au and can replace Au NPs for vis–NIR light absorption [147–148].

#### Bio-inspired plasmonic nanostructures/architectures

The pioneering works of several research groups have revealed that by mimicing biological systems, such as butterfly wings [149] and snake skin [150], systems can be designed that are cable of absorbing NIR light due to their distinctive nanoarchitectures with underlying mechanisms. For example, the velvet black scales on snake skins have been shown to demonstrate four times better absorbance than other scales in the UV–NIR spectrum range due to their hierarchical pattern of leaf-like microstructures with nanoridges [150]. In other case, the geometry of the scales of a butterfly promote a light trapping effect from the UV to NIR range, which significantly increases the light absorption compared to a non-patterned system [149]. These works inspired the combination of bismuth vanadate (BiVO_4_) with the 3D architecture design of a butterfly wing, in addition to gold nanorods (NRs) as plasmonic nanoantennas for an effective far red-to-NIR photocatalytic conversion. The electron–hole pairs were formed in some parts of BVO when BiVO_4_ encountered intense SPR-induced electric fields localized at Au NRs, and hence the entire photocatalytic system could function under red-to-NIR irradiation [151]. All these studies provide new insight into using plasmonic nanoarchitectures for photocatalysis applications in the future. Similarly, biomimetic assembly methods can also be used to arrange plasmonic metals (Au and Ag) with molecular-level precision to achieve tunable light absorption and emission. Prior works reported the use of DNA oligonucleotides [152–153] and virus capsids [154] as tunable spacers to control the distance between Au NPs and fluorophores. The assembly hierarchy of the plasmonic photocatalysts were carried out using both MS2 virus capsids and DNA origami as biological scaffolds to increase fluorescence intensity by tuning the distance between capsid and Au NPs [155]. In recent years, the phytochemicals present in plant-based and waste materials have been used as reducing and stabilizing agents to prepare plasmonic metals (Au and Ag). The so called green synthesis of Au and Ag are suitably used in clinical and biomedical fields because it is free of toxic chemicals and non-polar solvents. Numerous researches have proved that Au and Ag NPs can be synthesized from the chemicals extracted from plants and microorganisms such as fungi, algae, bacteria and yeasts [156–161]. Different types of biomolecules available in plants, for example, polysaccharides, phenolics, or flavonoids are capable of producing metal nanoparticles of different sizes and shapes [162]. This phytosynthesis is more favourable than that which occurs in microorganisms because it is fast and cost effective and can be readily scaled for realistic applications [163]. A very recent study demonstrated that Ag/AgCl can be synthesized using ash gourd peel extract without using organic toxic solvents [157]. Bovine serum albumin (BSA) and Neem extract were used to prepare Ag-ZnO nanostructures, in which both green extracts acted as a shape controllers and reducing agents of Ag^+^ to overcome the self-nucleation problem of Ag NPs [164–165].

#### Direct photocatalysis by plasmonic metals

Plasmonic catalyst systems have almost exclusively focused on the coupling of plasmonic metals (Au and Ag) with semiconductors. Recent reports found that plasmonic metals can be utilised to motivate direct photocatalysis where both light harvesting and activation of reactants take place on the plasmonic metals. It is observed that, unlike semiconductors, the photocatalytic quantum efficiency on plasmonic metal enhances the light intensity and thermal energy absorption. This result shows that plasmonic metals are effective at coupling thermal and photonic stimuli for driving chemical transformations [166]. The rapid recombination of plasmon charge carriers restricted the direct photocatalysis reaction on metal surfaces; however, the energy obtained as a result of recombination facilitated thermal reaction and resolved this drawback [167]. Since the foremost discovery of primary photocatalysis by Au NPs through LSPR [168], numerous reactions have been performed on Ag, Au, and Cu surfaces, showing that low intensity visible photon irradiation significantly enhances the rate of chemical reactions. A pioneering work showed that Au NPs have potential in degrading volatile organic compounds, HCHO to CO_2_, under 600–700 nm red light irradiation [168]. The same group also reported the use of Ag NPs to remove phenol and drive oxidation of benzyl alcohol to benzaldehyde under UV light. This verifies the role of the LSPR effect and interband transition of Ag NPs in activating organic molecules for oxidation under UV–vis irradiation [169]. Au NPs are more suitable for immediate plasmonic photocatalysis compared to other plasmonic metals because they are more chemically resistant and stable to standard atmosphere [170]. Under LED excitation at 530 nm in the presence of air or oxygen, Au NPs could promote the oxidation reaction of 9- anthraldehyde to form anthraquinone as the dominant product [171]. As compared to semiconductors like TiO_2_, the ability of Au NPs to conduct plasmonic-induced reductions at lower temperature and pressure enable the selection of unstable intermediates of a thermal reaction as the product (e.g., aromatic azo compounds). These findings show the potential of Au NPs in utilizing the solar spectrum, also in temperature-sensitive synthesis [172]. Most reported works on direct photocatalysis are limited only to plasmon-induced chemical transformations. Very few have moved away from this traditional route to demonstrate the feasibility of electrocatalytic oxidation adopting glucose accelerated by Au NPs upon LSPR excitation under a suitable voltage bias. The hot electrons injected from Au NPs can be driven into the external circuit to deliver appreciable current, while the holes facilitate the electrocatalytic oxidation of glucose owing to their equal energy levels. This constructive finding propelled the potential applications of electrochemical energy conversion, electroanalysis and electrochemical devices [173]. Overall, it is clear that the plasmonic metals are able to concentrate and channel the energy of low intensity visible light into adsorbed molecules to promote significant enhancement in the rate of chemical transformation. Furthermore, there are certain cases that have showed primary evidence for direct plasmon-driven photocatalysis capable of controlling catalytic selectivity through different reaction mechanisms. Gold NPs on CeO_2_ were found to be efficient at reducing a wide range of epoxides, azo compounds, and ketones at ambient temperature under visible light. Their reduction potential highly depends on the incident wavelength [174]. The team revealed the selectivity tuning by plasmon-mediated photo-switching and demonstrated the same for propylene epoxidation on Cu NPs in which the reduction of the Cu_2_O shell was brought on by the plasmon-excited Cu [175].

## Conclusion

This review explicitly detailed insight into plasmonic photocatalysts as a potential candidate for enhanced utilisation of the solar spectrum. The topic of the review was detailed through fundamental explanations together with the various synthesis routes. The review also clarified the mechanism of LSPR for the various noble metal nanoparticles in addition to the Schottky phenomenon on the studied metal oxide photocatalysts. An in-depth analysis on the formation and identification of ROSs and their interaction with pollutants was clearly presented. The future prospects of these sustainable photocatalysts with real-time applications for energy storage and environmental remediation were thoroughly reviewed. The present review also revealed the potential of plasmonic photocatalysts as an alternative sustainable approach and new direction for effectively harnessing sunlight to fulfil global environmental issues and aid to the energy crisis.

## References

[1] Zhou X, Liu G, Yu J, Fan W (2012). J Mater Chem.

[2] Ong W-J, Tan L-L, Chai S-P, Yong S-T, Mohamed A-R (2014). ChemSusChem.

[3] Kochuveedu S T, Jang Y H, Kim D H (2013). Chem Soc Rev.

[4] Chen H, Sun Z, Ni W, Woo K C, Lin H-Q, Sun L, Yan C, Wang J (2009). Small.

[5] Wang P, Huang B, Dai Y, Whangbo M-H (2012). Phys Chem Chem Phys.

[6] Zhang X, Chen Y L, Liu R-S, Tsai D P (2013). Rep Prog Phys.

[7] Wang Z, Liu Y, Huang B, Dai Y, Lou Z, Wang G, Zhang X, Qin X (2014). Phys Chem Chem Phys.

[8] Cushing S K, Li J, Meng F, Senty T R, Suri S, Zhi M, Li M, Bristow A D, Wu N (2012). J Am Chem Soc.

[9] Linic S, Christopher P, Ingram D B (2011). Nat Mater.

[10] Shin D O, Jeong J-R, Han T H, Koo C M, Park H-J, Lim Y T, Kim S O (2010). J Mater Chem.

[11] Moores A, Goettmann F (2006). New J Chem.

[12] Wang X, Zhu M, Sun Y, Fu W, Gu Q, Zhang C, Zhang Y, Dai Y, Sun Y (2016). Part Part Syst Charact.

[13] Sarina S, Waclawik E R, Zhu H (2013). Green Chem.

[14] Wang C, Astruc D (2014). Chem Soc Rev.

[15] Kelly K L, Coronado E, Zhao L L, Schatz G C (2003). J Phys Chem B.

[16] Clavero C (2014). Nat Photonics.

[17] Galian R E, Pérez-Prieto J (2010). Energy Environ Sci.

[18] Barnes W L, Dereux A, Ebbesen T W (2003). Nature.

[19] Chen Y, Xin X, Zhang N, Xu Y-J (2017). Part Part Syst Charact.

[20] Stahl S S (2005). Science.

[21] Boltasseva A, Atwater H A (2011). Science.

[22] Choi M, Shin K-H, Jang J (2010). J Colloid Interface Sci.

[23] Tada H, Kiyonaga T, Naya S-i (2009). Chem Soc Rev.

[24] Xie Y, Kum J, Zhao X, Cho S O (2011). Semicond Sci Technol.

[25] Mohapatra S K, Kondamudi N, Banerjee S, Misra M (2008). Langmuir.

[26] Kwak B-S, Chae J-H, Kim J-Y, Kang M-S (2009). Bull Korean Chem Soc.

[27] Garcia M A (2011). J Phys D: Appl Phys.

[28] Kale M J, Avanesian T, Christopher P (2014). ACS Catal.

[29] Molina R A, Weinmann D, Jalabert R A (2002). Phys Rev B.

[30] White T P, Catchpole K R (2012). Appl Phys Lett.

[31] Leong K H, Sim L C, Saravanan P, Ibrahim S, Ranjan S, Dasgupta N, Lichtfouse E (2016). Light Driven Nanomaterials for Removal of Agricultural Toxins. Nanoscience in Food and Agriculture 3.

[32] Bumajdad A, Madkour M (2014). Phys Chem Chem Phys.

[33] Qu Y, Duan X (2013). Chem Soc Rev.

[34] Liu S, Han C, Tang Z-R, Xu Y-J (2016). Mater Horiz.

[35] Wang H, Zhang L, Chen Z, Hu J, Li S, Wang Z, Liu J, Wang X (2014). Chem Soc Rev.

[36] Ong W-J, Tan L-L, Chai S-P, Yong S-T, Mohamed A-R (2014). Nanoscale.

[37] Wang J, Tang L, Zeng G, Liu Y, Zhou Y, Deng Y, Wang J, Peng B (2017). ACS Sustainable Chem Eng.

[38] Rajender G, Choudhury B, Giri P K (2017). Nanotechnology.

[39] Ong W-J, Putri L K, Tan L-L, Chai S-P, Yong S-T (2016). Appl Catal, B: Environ.

[40] Ong W-J, Putri L K, Tan Y-C, Tan L-L, Li N, Ng Y H, Wen X, Chai S-P (2017). Nano Res.

[41] Zeng D, Xu W, Ong W-J, Xu J, Ren H, Chen Y, Zheng H, Peng D-L (2018). Appl Catal, B: Environ.

[42] Zeng D, Ong W-J, Zheng H, Wu M, Chen Y, Peng D-L, Han M-Y (2017). J Mater Chem A.

[43] Ong W-J, Tan L-L, Ng Y H, Yong S-T, Chai S-P (2016). Chem Rev.

[44] Ong W-J (2017). Front Mater.

[45] Yu S, Wilson A J, Kumari G, Zhang X, Jain P K (2017). ACS Energy Lett.

[46] Rahmanian E, Malekfar R, Pumera M (2018). Chem – Eur J.

[47] Zeng D, Xiao L, Ong W-J, Wu P, Zheng H, Chen Y, Peng D-L (2017). ChemSusChem.

[48] Zhang Z, Zheng T, Li X, Xu J, Zeng H (2016). Part Part Syst Charact.

[49] Chen X, Li N, Kong Z, Ong W-J, Zhao X (2018). Mater Horiz.

[50] Leong K H, Chu H Y, Ibrahim S, Saravanan P (2015). Beilstein J Nanotechnol.

[51] Leong K H, Gan B L, Ibrahim S, Saravanan P (2014). Appl Surf Sci.

[52] Rather R A, Singh S, Pal B (2017). Sol Energy Mater Sol Cells.

[53] Singh J, Satpati B, Mohapatra S (2017). Plasmonics.

[54] Stucchi M, Bianchi C L, Argirusis C, Pifferi V, Neppolian B, Cerrato G, Boffito D C (2018). Ultrason Sonochem.

[55] Suwanchawalit C, Wongnawa S, Sriprang P, Meanha P (2012). Ceram Int.

[56] Zhao C, Zhu D-c, Cheng X-y, Cao S-x (2017). Front Mater Sci.

[57] Chen Z, Fang L, Dong W, Zheng F, Shen M, Wang J (2014). J Mater Chem A.

[58] Eom H, Jung J-Y, Shin Y, Kim S, Choi J-H, Lee E, Jeong J-H, Park I (2014). Nanoscale.

[59] Xia H, Wu S, Bi J, Zhang S (2017). Nanotechnology.

[60] Sung-Suh H M, Choi J R, Hah H J, Koo S M, Bae Y C J (2004). J Photochem Photobiol, A.

[61] Chen D, Chen Q, Ge L, Yin L, Fan B, Wang H, Lu H, Xu H, Zhang R, Shao G (2013). Appl Surf Sci.

[62] Kumar S, Kumar A, Bahuguna A, Sharma V, Krishnan V (2017). Beilstein J Nanotechnol.

[63] Xiang Q, Yu J, Cheng B, Ong H C (2010). Chem – Asian J.

[64] Bian Z, Tachikawa T, Zhang P, Fujitsuka M, Majima T (2014). J Am Chem Soc.

[65] Zhu X, Jin C, Li X-S, Liu J-L, Sun Z-G, Shi C, Li X, Zhu A-M (2017). ACS Catal.

[66] Tahir B, Tahir M, Amin N A S (2016). Clean Technol Environ Policy.

[67] Ding D, Liu K, He S, Gao C, Yin Y (2014). Nano Lett.

[68] Priebe J B, Radnik J, Lennox A J, Pohl M-M, Karnahl M, Hollmann D, Grabow K, Bentrup U, Junge H, Beller M (2015). ACS Catal.

[69] Bumajdad A, Madkour M, Abdel-Moneam Y, El-Kemary M (2014). J Mater Sci.

[70] Puga A V, Forneli A, García H, Corma A (2014). Adv Funct Mater.

[71] Mehta A, Sharma M, Kumar A, Basu S (2017). Gold Bull.

[72] Nalbandian M J, Greenstein K E, Shuai D, Zhang M, Choa Y-H, Parkin G F, Myung N V, Cwiertny D M (2015). Environ Sci Technol.

[73] Liu E, Fan J, Hu X, Hu Y, Li H, Tang C, Sun L, Wan J (2015). J Mater Sci.

[74] Khan M A, Sinatra L, Oufi M, Bakr O M, Idriss H (2017). Catal Lett.

[75] Seifvand N, Kowsari E (2016). Ind Eng Chem Res.

[76] Kalarivalappil V, Divya C M, Wunderlich W, Pillai S C, Hinder S J, Nageri M, Kumar V, Vijayan B K (2016). Catal Lett.

[77] Li N, Liu M, Yang B, Shu W, Shen Q, Liu M, Zhou J (2017). J Phys Chem C.

[78] Fujimoto T M, Ponczek M, Rochetto U L, Landers R, Tomaz E (2017). Environ Sci Pollut Res.

[79] Wu J, Lu S, Ge D, Zhang L, Chen W, Gu H (2016). RSC Adv.

[80] Yilmaz P, Lacerda A M, Larrosa I, Dunn S (2017). Electrochim Acta.

[81] Ong W-J, Tan L-L, Chai S-P, Yong S-T (2015). Dalton Trans.

[82] Zhu Z, Wu R-J (2015). J Taiwan Inst Chem Eng.

[83] Shiraishi Y, Sakamoto H, Fujiwara K, Ichikawa S, Hirai T (2014). ACS Catal.

[84] Wei P, Liu J, Li Z (2013). Ceram Int.

[85] Wang W-K, Chen J-J, Li W-W, Pei D-N, Zhang X, Yu H-Q (2015). ACS Appl Mater Interfaces.

[86] Lv J, Gao H, Wang H, Lu X, Xu G, Wang D, Chen Z, Zhang X, Zheng Z, Wu Y (2015). Appl Surf Sci.

[87] Putri L K, Ong W-J, Chang W S, Chai S-P (2016). Catal Sci Technol.

[88] Ingram D B, Linic S (2011). J Am Chem Soc.

[89] Thomann I, Pinaud B A, Chen Z, Clemens B M, Jaramillo T F, Brongersma M L (2011). Nano Lett.

[90] Torimoto T, Horibe H, Kameyama T, Okazaki K-i, Ikeda S, Matsumura M, Ishikawa A, Ishihara H (2011). J Phys Chem Lett.

[91] Chai S Y, Kim Y J, Jung M H, Chakraborty A K, Jung D, Lee W I (2009). J Catal.

[92] Wang C, Shao C, Liu Y, Zhang L (2008). Scr Mater.

[93] Sun S, Wang W, Zhang L, Shang M, Wang L (2009). Catal Commun.

[94] Christopher P, Xin H, Linic S (2011). Nat Chem.

[95] Wu T, Liu S, Luo Y, Lu W, Wang L, Sun X (2011). Nanoscale.

[96] Chen X, Zhu H-Y, Zhao J-C, Zheng Z-F, Gao X-P (2008). Angew Chem, Int Ed Engl.

[97] Langhammer C, Yuan Z, Zorić I, Kasemo B (2006). Nano Lett.

[98] Langhammer C, Kasemo B, Zorić I (2007). J Chem Phys.

[99] Tabor C, Murali R, Mahmoud M, El-Sayed M A (2009). J Phys Chem A.

[100] Zhang Q, Tan Y N, Xie J, Lee J Y (2009). Plasmonics.

[101] Xu H, Li H, Xia J, Yin S, Luo Z, Liu L, Xu L (2011). ACS Appl Mater Interfaces.

[102] Wang P, Huang B, Zhang X, Qin X, Jin H, Dai Y, Wang Z, Wei J, Zhan J, Wang S (2009). Chem – Eur J.

[103] Purbia R, Paria S (2017). Dalton Trans.

[104] Shuang S, Lv R, Xie Z, Zhang Z (2016). Sci Rep.

[105] Sá J, Tagliabue G, Friedli P, Szlachetko J, Rittmann-Frank M H, Santomauro F G, Milne C J, Sigg H (2013). Energy Environ Sci.

[106] Amidani L, Naldoni A, Malvestuto M, Marelli M, Glatzel P, Dal Santo V, Boscherini F (2015). Angew Chem.

[107] Furube A, Du L, Hara K, Katoh R, Tachiya M (2007). J Am Chem Soc.

[108] Chen H M, Chen C K, Chen C-J, Cheng L-C, Wu P C, Cheng B H, Ho Y Z, Tseng M L, Hsu Y-Y, Chan T-S (2012). ACS Nano.

[109] Wei Z, Rosa L, Wang K, Endo M, Juodkazis S, Ohtani B, Kowalska E (2017). Appl Catal, B.

[110] Zhang L, Herrmann L O, Baumberg J J (2015). Sci Rep.

[111] Cui J, Li Y, Liu L, Chen L, Xu J, Ma J, Fang G, Zhu E, Wu H, Zhao L (2015). Nano Lett.

[112] Yang W, Xiong Y, Zou L, Zou Z, Li D, Mi Q, Wang Y, Yang H (2016). Nanoscale Res Lett.

[113] Ohno T, Masaki Y, Hirayama S, Matsumura M (2001). J Catal.

[114] Nakamura R, Nakato Y (2004). J Am Chem Soc.

[115] Nosaka Y, Nosaka Y A (2017). Chem Rev.

[116] Diesen V, Jonsson M (2014). J Phys Chem C.

[117] Hirakawa T, Koga C, Negishi N, Takeuchi K, Matsuzawa S (2009). Appl Catal, B.

[118] Sahel K, Elsellami L, Mirali I, Dappozze F, Bouhent M, Guillard C (2016). Appl Catal, B.

[119] Wu T, Liu G, Zhao J, Hidaka H, Serpone N (1999). J Phys Chem B.

[120] Chen C, Lei P, Ji H, Ma W, Zhao J, Hidaka H, Serpone N (2004). Environ Sci Technol.

[121] Lippert A R, Van De Bittner G C, Chang C J (2011). Acc Chem Res.

[122] Fu X, Tang Y, Dickinson B C, Chang C J, Chang Z (2015). Biochem Biophys Res Commun.

[123] Wang D, Zhao L, Guo L-H, Zhang H (2014). Anal Chem.

[124] Nakamura K, Ishiyama K, Ikai H, Kanno T, Sasaki K, Niwano Y, Kohno M (2011). J Clin Biochem Nutr.

[125] Tachikawa T, Majima T (2007). J Fluoresc.

[126] Tachikawa T, Majima T (2010). Chem Soc Rev.

[127] Yagi M, Takemoto S, Sasase R (2004). Chem Lett.

[128] Murakami Y, Endo K, Ohta I, Nosaka A Y, Nosaka Y (2007). J Phys Chem C.

[129] Sawada T, Yoshino F, Kimoto K, Takahashi Y, Shibata T, Hamada N, Sawada T, Toyoda M, Lee M-C (2010). J Dent Res.

[130] Naito K, Tachikawa T, Fujitsuka M, Majima T (2008). J Phys Chem C.

[131] Kim W, Tachikawa T, Moon G-h, Majima T, Choi W (2014). Angew Chem, Int Ed.

[132] Hawkins C L, Davies M J (2014). Biochim Biophys Acta, Gen Subj.

[133] Zhao H, Joseph J, Zhang H, Karoui H, Kalyanaraman B (2001). Free Radical Biol Med.

[134] Saita M, Kobatashi K, Yoshino F, Hase H, Nonami T, Kimoto K, Lee M-C-i (2012). Dent Mater J.

[135] Zhou P, Yu J, Jaroniec M (2014). Adv Mater.

[136] Kittel C (1976). Introduction to Solid State Physics.

[137] Wu X, Yin S, Dong Q, Liu B, Wang Y, Sekino T, Lee S W, Sato T (2013). Sci Rep.

[138] Manthiram K, Alivisatos A P (2012). J Am Chem Soc.

[139] Zhao Y, Burda C (2012). Energy Environ Sci.

[140] Naik G V, Shalaev V M, Boltasseva A (2013). Adv Mater.

[141] Zhou D, Liu D, Xu W, Yin Z, Chen X, Zhou P, Cui S, Chen Z, Song H (2016). ACS Nano.

[142] Kriegel I, Jiang C, Rodríguez-Fernandez J, Schaller R D, Talapin D V, Da Como E, Feldmann J (2012). J Am Chem Soc.

[143] Dorfs D, Härtling T, Miszta K, Bigall N C, Kim M R, Genovese A, Falqui A, Povia M, Manna L (2011). J Am Chem Soc.

[144] Mendelsberg R J, Lim S H N, Zhu Y K, Wallig J, Milliron D J, Anders A (2011). J Phys D: Appl Phys.

[145] Zhang Z, Huang J, Fang Y, Zhang M, Liu K, Dong B (2017). Adv Mater.

[146] Li J, Li W, Li X, Li Y, Bai H, Li M, Xi G (2017). RSC Adv.

[147] Naik G V, Schroeder J L, Ni X, Kildishev A V, Sands T D, Boltasseva A (2012). Opt Mater Express.

[148] Guler U, Kildishev A V, Boltasseva A, Shalaev V M (2015). Faraday Discuss.

[149] Van Hooijdonk E, Vandenbem C, Berthier S, Vigneron J P (2012). Opt Express.

[150] Spinner M, Kovalev A, Gorb S N, Westhoff G (2013). Sci Rep.

[151] Yan R, Chen M, Zhou H, Liu T, Tang X, Zhang K, Zhu H, Ye J, Zhang D, Fan T (2016). Sci Rep.

[152] Chhabra R, Sharma J, Wang H N, Zou S L, Lin S, Yan H, Lindsay S, Liu Y (2009). Nanotechnology.

[153] Cheng W, Campolongo M J, Cha J J, Tan S J, Umbach C C, Muller D A, Luo D (2009). Nat Mater.

[154] Capehart S L, Coyle M P, Glasgow J E, Francis M B (2013). J Am Chem Soc.

[155] Wang D, Capehart S L, Pal S, Liu M, Zhang L, Schuck P J, Liu Y, Yan H, Francis M B, De Yoreo J J (2014). ACS Nano.

[156] Akhtar M S, Panwar J, Yun Y-S (2013). ACS Sustainable Chem Eng.

[157] Devi T B, Ahmaruzzaman M (2016). Environ Sci Pollut Res.

[158] Huang J, Li Q, Sun D, Lu Y, Su Y, Yang X, Wang H, Wang Y, Shao W, He N (2007). Nanotechnology.

[159] Joerger R, Klaus T, Granqvist C G (2000). Adv Mater.

[160] Bhainsa K C, D'souza S F (2006). Colloids Surf, B.

[161] Gardea-Torresdey J L, Parsons J G, Gomez E, Peralta-Videa J, Troiani H E, Santiago P, Yacaman M J (2002). Nano Lett.

[162] Palanco M E, Mogensen K B, Gühlke M, Heiner Z, Kneipp J, Kneipp K (2016). Beilstein J Nanotechnol.

[163] Shankar S S, Rai A, Ahmad A, Sastry M (2004). J Colloid Interface Sci.

[164] Gao S, Jia X, Yang S, Li Z, Jiang K (2011). J Solid State Chem.

[165] Patil S S, Mali M G, Tamboli M S, Patil D R, Kulkarni M V, Yoon H, Kim H, Al-Deyab S S, Yoon S S, Kolekar S S (2016). Catal Today.

[166] Christopher P, Xin H, Marimuthu A, Linic S (2012). Nat Mater.

[167] Xiao Q, Jaatinen E, Zhu H (2014). Chem – Asian J.

[168] Chen X, Zhu H-Y, Zhao J-C, Zheng Z-F, Gao X-P (2008). Angew Chem.

[169] Chen X, Zheng Z, Ke X, Jaatinen E, Xie T, Wang D, Guo C, Zhao J, Zhu H (2010). Green Chem.

[170] Cheng H, Fuku K, Kuwahara Y, Mori K, Yamashita H (2015). J Mater Chem A.

[171] Wee T-L, Schmidt L C, Scaiano J C (2012). J Phys Chem C.

[172] Zhu H, Ke X, Yang X, Sarina S, Liu H (2010). Angew Chem.

[173] Wang C, Nie X-G, Shi Y, Zhou Y, Xu J-J, Xia X-H, Chen H-Y (2017). ACS Nano.

[174] Ke X, Sarina S, Zhao J, Zhang X, Chang J, Zhu H (2012). Chem Commun.

[175] Marimuthu A, Zhang J, Linic S (2013). Science.

